# Gut bacteriome and mood disorders in women with PCOS

**DOI:** 10.1093/humrep/deae073

**Published:** 2024-04-13

**Authors:** S Lee, M V Tejesvi, E Hurskainen, O Aasmets, J Plaza-Díaz, S Franks, L Morin-Papunen, J S Tapanainen, T S Ruuska, S Altmäe, E Org, A Salumets, R K Arffman, T T Piltonen

**Affiliations:** Department of Obstetrics and Gynecology, Research Unit of Clinical Medicine, Medical Research Center, Oulu University Hospital, University of Oulu, Oulu, Finland; Department of Obstetrics and Gynaecology, Institute of Clinical Medicine, University of Tartu, Tartu, Estonia; Research Unit of Clinical Medicine, University of Oulu, Oulu, Finland; Ecology and Genetics, University of Oulu, Oulu, Finland; Department of Obstetrics and Gynecology, Research Unit of Clinical Medicine, Medical Research Center, Oulu University Hospital, University of Oulu, Oulu, Finland; Estonian Genome Centre, Institute of Genomics, University of Tartu, Tartu, Estonia; Faculty of Pharmacy, Department of Biochemistry and Molecular Biology II, University of Granada, Granada, Spain; Instituto de Investigación Biosanitaria ibs. GRANADA, Granada, Spain; Children’s Hospital of Eastern Ontario Research Institute, Ottawa, Canada; Department of Metabolism, Digestion and Reproduction, Faculty of Medicine, Imperial College London, London, UK; Department of Obstetrics and Gynecology, Research Unit of Clinical Medicine, Medical Research Center, Oulu University Hospital, University of Oulu, Oulu, Finland; Department of Obstetrics and Gynecology, Research Unit of Clinical Medicine, Medical Research Center, Oulu University Hospital, University of Oulu, Oulu, Finland; Department of Obstetrics and Gynecology, University of Helsinki and Helsinki University Hospital, Helsinki, Finland; Department of Obstetrics and Gynaecology, HFR—Cantonal Hospital of and University of Fribourg, Fribourg, Switzerland; Research Unit of Clinical Medicine, University of Oulu, Oulu, Finland; Department of Pediatrics and Adolescent Medicine and Medical Research Center, Oulu University Hospital, Oulu, Finland; Faculty of Pharmacy, Department of Biochemistry and Molecular Biology II, University of Granada, Granada, Spain; Instituto de Investigación Biosanitaria ibs. GRANADA, Granada, Spain; Division of Obstetrics and Gynaecology, Department of Clinical Science, Intervention and Technology (CLINTEC), Karolinska Institute and Karolinska University Hospital, Stockholm, Sweden; Estonian Genome Centre, Institute of Genomics, University of Tartu, Tartu, Estonia; Department of Obstetrics and Gynaecology, Institute of Clinical Medicine, University of Tartu, Tartu, Estonia; Division of Obstetrics and Gynaecology, Department of Clinical Science, Intervention and Technology (CLINTEC), Karolinska Institute and Karolinska University Hospital, Stockholm, Sweden; Competence Centre on Health Technologies, Tartu, Estonia; Department of Obstetrics and Gynecology, Research Unit of Clinical Medicine, Medical Research Center, Oulu University Hospital, University of Oulu, Oulu, Finland; Department of Obstetrics and Gynecology, Research Unit of Clinical Medicine, Medical Research Center, Oulu University Hospital, University of Oulu, Oulu, Finland

**Keywords:** gut microbiome, gut bacteriome, mood disorders, anxiety, depression, polycystic ovary syndrome, PCOS

## Abstract

**STUDY QUESTION:**

How does the gut bacteriome differ based on mood disorders (MDs) in women with polycystic ovary syndrome (PCOS), and how can the gut bacteriome contribute to the associations between these two conditions?

**SUMMARY ANSWER:**

Women with PCOS who also have MDs exhibited a distinct gut bacteriome with reduced alpha diversity and a significantly lower abundance of *Butyricicoccus* compared to women with PCOS but without MDs.

**WHAT IS KNOWN ALREADY:**

Women with PCOS have a 4- to 5-fold higher risk of having MDs compared to women without PCOS. The gut bacteriome has been suggested to influence the pathophysiology of both PCOS and MDs.

**STUDY DESIGN, SIZE, DURATION:**

This population-based cohort study was derived from the Northern Finland Birth Cohort 1966 (NFBC1966), which includes all women born in Northern Finland in 1966. Women with PCOS who donated a stool sample at age 46 years (n = 102) and two BMI-matched controls for each case (n = 205), who also responded properly to the MD criteria scales, were included.

**PARTICIPANTS/MATERIALS, SETTING, METHODS:**

A total of 102 women with PCOS and 205 age- and BMI-matched women without PCOS were included. Based on the validated MD criteria, the subjects were categorized into MD or no-MD groups, resulting in the following subgroups: PCOS no-MD (n = 84), PCOS MD (n = 18), control no-MD (n = 180), and control MD (n = 25). Clinical characteristics were assessed at age 31 years and age 46 years, and stool samples were collected from the women at age 46 years, followed by the gut bacteriome analysis using 16 s rRNA sequencing. Alpha diversity was assessed using observed features and Shannon’s index, with a focus on genera, and beta diversity was characterized using principal components analysis (PCA) with Bray–Curtis Dissimilarity at the genus level. Associations between the gut bacteriome and PCOS-related clinical features were explored by Spearman’s correlation coefficient. A *P*-value for multiple testing was adjusted with the Benjamini–Hochberg false discovery rate (FDR) method.

**MAIN RESULTS AND THE ROLE OF CHANCE:**

We observed changes in the gut bacteriome associated with MDs, irrespective of whether the women also had PCOS. Similarly, PCOS MD cases showed a lower alpha diversity (Observed feature, PCOS no-MD, median 272; PCOS MD, median 208, FDR = 0.01; Shannon, PCOS no-MD, median 5.95; PCOS MD, median 5.57, FDR = 0.01) but also a lower abundance of *Butyricicoccus* (log-fold change_Analysis of Compositions of Microbiomes with Bias Correction (ANCOM-BC)_=−0.90, FDR_ANCOM-BC_=0.04) compared to PCOS no-MD cases. In contrast, in the controls, the gut bacteriome did not differ based on MDs. Furthermore, in the PCOS group, *Sutterella* showed positive correlations with PCOS-related clinical parameters linked to obesity (BMI, *r*^2^=0.31, FDR = 0.01; waist circumference, *r*^2^=0.29, FDR = 0.02), glucose metabolism (fasting glucose, *r*^2^=0.46, FDR < 0.001; fasting insulin, *r*^2^=0.24, FDR = 0.05), and gut barrier integrity (zonulin, *r*^2^=0.25, FDR = 0.03).

**LIMITATIONS, REASONS FOR CAUTION:**

Although this was the first study to assess the link between the gut bacteriome and MDs in PCOS and included the largest PCOS dataset for the gut microbiome analysis, the number of subjects stratified by the presence of MDs was limited when contrasted with previous studies that focused on MDs in a non-selected population.

**WIDER IMPLICATIONS OF THE FINDINGS:**

The main finding is that gut bacteriome is associated with MDs irrespective of the PCOS status, but PCOS may also modulate further the connection between the gut bacteriome and MDs.

**STUDY FUNDING/COMPETING INTEREST(S):**

This research was funded by the European Union’s Horizon 2020 Research and Innovation Programme under the Marie Sklodowska-Curie Grant Agreement (MATER, No. 813707), the Academy of Finland (project grants 315921, 321763, 336449), the Sigrid Jusélius Foundation, Novo Nordisk Foundation (NNF21OC0070372), grant numbers PID2021-12728OB-100 (Endo-Map) and CNS2022-135999 (ROSY) funded by MCIN/AEI/10.13039/501100011033 and ERFD A Way of Making Europe. The study was also supported by EU QLG1-CT-2000-01643 (EUROBLCS) (E51560), NorFA (731, 20056, 30167), USA/NIH 2000 G DF682 (50945), the Estonian Research Council (PRG1076, PRG1414), EMBO Installation (3573), and Horizon 2020 Innovation Grant (ERIN, No. EU952516). The funders did not participate in any process of the study. We have no conflicts of interest to declare.

**TRIAL REGISTRATION NUMBER:**

N/A.

## Introduction

Polycystic ovary syndrome (PCOS), which affects one out of eight women, can be diagnosed when at least two of the following symptoms are present according to the Rotterdam consensus criteria: oligo- or anovulation (OA), biochemical or clinical hyperandrogenism (HA), and polycystic ovarian morphology (PCOM), with the exclusion of other androgen excess-related disorders (‘Revised 2003 consensus on diagnostic criteria and long-term health risks related to polycystic ovary syndrome (PCOS)’, 2004; Azziz *et al.*, 2006; Teede *et al.*, 2023). The endocrine abnormalities and chronic inflammation observed in PCOS, represented by increased inflammatory markers (e.g. C-reactive protein (CRP), interleukin-6 (IL-6), and tumor necrosis factor-α (TNF-α)) (Aboeldalyl *et al.*, 2021), are not only associated with metabolic derangements, such as obesity and insulin resistance (IR) (Escobar-Morreale, 2018), but also elevate the risk of mood disorders (MDs), which have been shown to be 4–5 times more common in women with PCOS compared to other women (Cooney *et al.*, 2017; Karjula *et al.*, 2017, 2021; Kolhe *et al.*, 2022). The etiology of MDs in PCOS is complex and multifactorial (Karjula *et al.*, 2017, 2021; Kolhe *et al.*, 2022).

The gut microbiome, particularly the bacterial community known as the bacteriome, communicates with its host by influencing immune maturation and regulation (Cerf-Bensussan and Gaboriau-Routhiau, 2010; Hooper and Macpherson, 2010), sex hormone metabolism (He *et al.*, 2021), metabolic processes (Devaraj *et al.*, 2013), and even brain function (Arneth, 2018; Peirce and Alviña, 2019; Simpson *et al.*, 2021). Changes in the gut bacteriome of women with PCOS, exemplified by lower alpha diversity (Jobira *et al.*, 2020; Liang *et al.*, 2020; García-Bernal *et al.*, 2021; Zhu *et al.*, 2021) and shifts in the abundance of specific bacteria (e.g. *Bacteroides, Escherichia/Shigella*) (Liu *et al.*, 2017; Zhang *et al.*, 2019; Chu *et al.*, 2020; Haudum *et al.*, 2020; Chen *et al.*, 2021; Zhu *et al.*, 2021) compared to non-PCOS women, can impact the pathogenesis of PCOS through several intertwined mechanisms. First, the altered gut bacteriome can elevate circulating androgens via beta-glucuronidase enzyme-driven deconjugation (Pellock and Redinbo, 2017; Batra *et al.*, 2022). Second, the altered bacterial composition can trigger systemic low-grade inflammation by compromising gut barrier integrity (Ye *et al.*, 2006; Swidsinski *et al.*, 2007; Tremellen and Pearce, 2012), promoting insulin resistance (Caricilli and Saad, 2013) and HA (He and Li, 2020). Additionally, changes in the gut bacteriome can influence gut hormone secretion, including ghrelin and peptide YY, which influence pituitary function and further exaggerate the sex hormone imbalance in PCOS (Sola-Leyva *et al.*, 2023). Furthermore, the alterations in the gut bacteriome can influence anxiety and depression, collectively referred to here as MDs, by (i) regulating the synthesis of neurotransmitters and their precursors, such as serotonin, gamma-aminobutyric acid, and tryptophan, (ii) altering neuropeptide and gut hormone release, as well as brain-derived neurotrophic factor, and (iii) promoting neuroinflammation (Peirce and Alviña, 2019; Simpson *et al.*, 2021). Given the involvement of the gut bacteriome in the pathophysiologies of both PCOS and MDs, it is important to understand its associations with MDs in women with PCOS.

Our main hypothesis was that women with PCOS who also had MDs exhibited an altered gut bacteriome compared to women with PCOS but without MDs, as well as non-PCOS women with MDs. By using 102 women with PCOS (84 PCOS no-MD, 18 PCOS MD) and 205 BMI-matched women without PCOS (180 control no-MD, 25 control MD), as a comparator group, our objectives were to (i) characterize the gut bacteriome profile in relation to MDs in women with PCOS and in controls and, (ii) investigate the associations between the gut bacteriome and PCOS-related clinical parameters, with the ultimate goal of elucidating possible facilitating factors between PCOS and MDs.

## Materials and methods

### Study population

#### Northern Finland Birth Cohort

The study population was derived from the Northern Finland Birth Cohort 1966 (NFBC1966) dataset, a longitudinal cohort of individuals born in 1966 in Northern Finland. The NFBC1966 subjects have been followed since birth and have been monitored throughout their lives (Rantakallio, 1988). Detailed study protocols, questionnaire forms, data coverage, and related articles can be accessed on the cohort website (www.oulu.fi/nfbc).

#### PCOS and control samples

The study population included in the current study has been described previously (Lüll *et al.*, 2021). Briefly, the PCOS group was composed of women who either reported PCOS symptoms at 31 years (OA and HA) (n = 125) or a history of PCOS at 46 years (reported PCOM/PCOS diagnosis) (n = 181). To ascertain the exclusive associations between the gut bacteriome and MDs within the PCOS context, a control group was included, comprising individuals who reported neither OA nor HA at 31 years and no PCOM/PCOS at 46 years (n = 1,573). Participants who were pregnant or using hormonal contraceptives at 31 years were excluded (n = 1,488), as well as those who had used antibiotics, antimycotics, or tamoxifen within three months prior to stool sample collection (n = 849). All women with PCOS who had donated a stool sample at age 46 years were identified, and two BMI-matched controls were selected for each case. Therefore, the final sample size consisted of 102 women with PCOS and 205 women without PCOS. The study flow chart is shown in Supplementary Fig. S1. The study was approved by the Ethics Committee of the Northern Ostrobothnia Hospital District (EETTMK 94/2011), and informed consent was signed by all cohort participants in this study.

#### MD assessment

At age 46 years, MDs, including anxiety and depression, were assessed using the following four criteria: Beck Depression Inventory Second Edition (BDI-II), Generalized Anxiety Disorder Assessment (GAD-7), Hopkins Symptom Checklist (HSCL)-25, and a self-reported diagnosis with depression (Supplementary Table S1) (Guze, 1995; Beck *et al.*, 1996). Women meeting at least two out of the four criteria were classified as the MD group (Sinikumpu *et al.*, 2023). Four participants who did not properly report the MD criteria scales were excluded (Supplementary Fig. S1). To confirm previous findings demonstrating changes in the gut bacteriome related to MDs (Simpson *et al.*, 2021) using our population, the subjects were grouped based on MDs irrespective of PCOS status (no-MD total n = 264, MD total n = 43). Following this, the subjects were classified based on both PCOS and MD status. Therefore, the final sample size consisted of 84 PCOS no-MD, 18 PCOS with MD, 180 control no-MD, and 25 control with MD.

### Baseline characteristic measurements

#### Anthropometric measurements

BMI (kg/m^2^) was calculated by dividing weight (kg) by height squared (m^2^), and waist circumference (cm) was measured at the midpoint between the lowest rib and the iliac crest.

#### Sex hormone measurements

Serum testosterone (T) and sex hormone-binding globulin (SHBG) were measured using Agilent triple quadrupole 6410 liquid chromatography/mass spectrometry equipment (Agilent Technologies, Wilmington, DE, USA) and chemiluminometric immunoassay (Immulite 2000, Siemens Health Care, Llanberis, UK), respectively. The free androgen index (FAI) was calculated by dividing serum T (nmol/l) by SHBG (nmol/l), then multiplying by 100.

#### Glucose metabolism

Plasma glucose was analyzed using an enzymatic dehydrogenase method (Advia 1800, Siemens Healthcare Diagnostics, UK), and serum insulin was measured by a chemiluminometric immunoassay (Advia Centaur XP, Siemens Healthcare Diagnostics, UK). IR was assessed using the homeostasis model assessment of insulin resistance (HOMA-IR), calculated as fasting serum insulin [µU/ml]×fasting plasma glucose [mmol/l]/22.5 (Matthews *et al.*, 1985).

#### Inflammatory and gut permeability markers

Serum levels of high-sensitivity CRP (hs-CRP) were measured by nephelometric assay (BN ProSpec, Siemens Healthcare Diagnostics, UK). Zonulin and intestinal fatty acid-binding protein-2 (FABP2) levels in the serum were measured using ELISA (R&D Systems, Minneapolis, MN, USA, and Immundiagnostik AG, Bensheim, Germany) according to the manufacturer’s instructions. The above clinical parameters of the study participants are summarized in Tables 1 and 2.

**Table 1.** Baseline characteristics of the no-MD and MD cases in the whole population at age 46 years.

	no-MD	MD	FDR
BMI, kg/m^2^	27.74 [5.40] (264)	28.82 [5.75] (43)	0.25
Waist circumference, cm	89.16 [12.69] (263)	94.84 [14.75] (42)	**0.02**
T, nmol/l	0.85 [0.11] (263)	0.94 [0.11] (43)	0.42
SHBG, nmol/l	51.70 [11.80; 224.00] (262)	44.20 [14.00; 157.00] (43)	0.16
FAI	1.59 [0.03; 9.15] (262)	1.89 [0.04; 6.64] (43)	0.33
HOMA-IR index	1.82 [0.50; 11.04] (256)	2.15 [0.37; 58.20] (42)	0.07
Fasting glucose, mmol/l	5.30 [4.20; 8.30] (258)	5.30 [4.40; 6.20] (42)	0.51
Fasting insulin, mU/I	7.65 [5.48; 11.13] (258)	9.20 [6.05; 15.23] (42)	0.08
hs-CRP, mg/l	0.83 [0.17; 17.30] (249)	0.92 [0.20; 19.10] (42)	0.92
Zonulin, ng/ml	128.26 [16.00] (262)	123.23 [16.29] (43)	0.07
FABP2, ng/ml	1.25 [0.34; 6.01] (262)	1.58 [0.39; 6.40] (43)	**0.02**

Demographic characteristics are presented as mean with [SD] or median with interquartile range [Q1; Q3] based on data distribution and normality. The number of subjects is indicated in parentheses. *P*-value was determined by the *t*-test or Mann–Whitney *U*-test with the Benjamini–Hochberg FDR adjustment, and bold values represent FDR < 0.05.

MD, mood disorder; FDR, false discovery rate; T, testosterone; SHBG, sex hormone-binding globulin; FAI, free androgen index; HOMA-IR, homeostatic model assessment for insulin resistance; hs-CRP, high-sensitive C-reactive protein; FABP2, fatty acid-binding protein 2.

**Table 2.** Baseline characteristics of the no-MD and MD cases in the PCOS and control groups at age 46 years.

	PCOS		Control		FDR^a^		FDR^b^	
	no-MD	MD	no-MD	MD	PCOS	Control	no-MD	MD
BMI, kg/m^2^	27.54 [5.24] (84)	29.22 [6.46] (18)	27.84 [5.48] (180)	28.52 [5.30] (25)	0.24	0.56	0.68	0.70
Waist circumference, cm	88.38 [12.03] (83)	95.91 [16.89] (18)	89.52 [13.00] (180)	94.04 [13.24] (24)	0.09	0.11	0.50	0.69
T, nmol/l	0.94 [0.33] (84)	0.91 [0.32] (18)	0.84 [0.33] (179)	0.90 [0.38] (25)	0.73	0.41	**0.03**	0.95
SHBG, nmol/l	51.05 [34.73; 72.05] (84)	49.00 [29.23; 63.03] (18)	53.80 [38.58; 73.08] (178)	43.30 [29.85; 70.15] (25)	0.41	0.22	0.74	0.94
FAI	1.75 [1.40; 2.59] (84)	1.72 [1.33; 3.24] (18)	1.49 [1.14; 2.07] (178)	1.98 [1.06; 2.71] (25)	0.97	0.40	**0.01**	0.77
HOMA-IR index	1.80 [1.27; 2.71] (82)	2.80 [1.50; 4.78] (18)	1.82 [1.22; 2.72] (174)	1.98 [1.14; 3.47] (24)	**0.03**	0.63	0.94	0.17
Fasting glucose, mmol/l	5.30 [5.10; 5.70] (83)	5.65 [5.20; 5.93] (18)	5.30 [5.10; 5.70] (173)	5.30 [5.00; 5.50] (23)	**0.01**	0.98	1.00	**0.03**
Fasting insulin, mU/I	7.45 [5.58; 11.23] (82)	11.45 [6.48; 16.78] (18)	7.80 [5.33; 11.10] (176)	8.25 [5.13; 14.73] (24)	**0.04**	0.61	0.99	0.18
hs-CRP, mg/l	0.86 [0.17; 8.98] (82)	0.84 [0.22; 19.10] (17)	0.79 [0.16; 17.30] (175)	0.78 [0.20; 6.51] (24)	0.37	0.52	0.52	0.41
Zonulin, ng/ml	129.71 [14.23] (84)	135.30 [12.44] (18)	127.57 [16.77] (178)	130.02 [18.52] (25)	0.13	0.50	0.31	0.30
FABP2, ng/ml	1.31 [0.44; 4.18] (84)	1.74 [0.62; 4.08] (18)	1.29 [0.34; 6.01] (178)	2.17 [0.39; 6.40] (25)	0.13	0.10	0.76	0.96

Demographic characteristics are presented as mean with [SD] or median with interquartile range [Q1; Q3] based on data distribution and normality. The number of subjects is indicated in parentheses. *P*-value was determined by the *t*-test or Mann–Whitney *U*-test with the Benjamini–Hochberg FDR adjustment. ^a^FDR is the statistical analysis between no-MD and MD cases in each group (PCOS or control), and ^b^FDR is the statistical analysis between PCOS and control cases with the same mood disorder status. Bold values represent FDR < 0.05.

MD, mood disorder; FDR, false discovery rate; T, testosterone; SHBG, sex hormone-binding globulin; FAI, free androgen index; HOMA-IR, homeostatic model assessment for insulin resistance; hs-CRP, high-sensitive C-reactive protein; FABP2, fatty acid-binding protein 2.

### Gut bacteriome analysis

#### Gut bacterial DNA extraction and 16S rRNA sequencing data analysis

The stool samples were collected in 2012 and stored at −80°C until 2019, when the bacterial DNA was extracted (QIAamp Stool Mini Kit, Qiagen, Venlo, The Netherlands) and sequenced using the V3–V4 regions of the 16S rRNA gene on an Illumina MiSeq sequencing instrument. Further details of the DNA extraction protocol and polymerase chain reaction conditions can be found in our previous study (Lüll *et al.*, 2021). Quantitative Insights Into Microbial Ecology 2 (QIIME2, version 2021.4) was used to analyze the sequencing data (Bolyen *et al.*, 2019). The reads were demultiplexed and denoised using DADA2 (Callahan *et al.*, 2016). The q2-dada2-denoise script was used to truncate the forward reads at position 285 and to trim at position 16 after retrieving the quality scores. Reverse reads were not trimmed; instead, they were truncated at position 240. Chimeras were removed using the q2-dada2-denoise’s ‘consensus’ filter. A phylogenetic tree was drawn using fasttree2 (Price *et al.*, 2010), and taxonomic classification was assigned using the SILVA database (version 138) (Quast *et al.*, 2012). Specific reads from cyanobacteria, mitochondria, eukaryota, and archaea were also removed from the final dataset. Ultimately, 4,207 amplified sequence variants (ASVs) were identified as belonging to 253 genera, 86 families, 48 orders, 21 classes, and 11 phyla. Taxa present in <30% of the samples were filtered out to focus on more common taxa and reduce the burden of multiple testing, resulting in 251 ASVs across 111 genera, 45 families, 28 orders, 13 classes, and 7 phyla.

### Statistical analysis

#### Clinical parameters

The baseline characteristics of the study subjects were analyzed using IBM SPSS Statistics version 27 (IBM Corporation, Armonk, NY, USA). For continuous variables, statistical differences were analyzed using independent samples *t*-test or the Mann–Whitney *U*-test for paired comparisons, and the Kruskal–Wallis test was used for multiple comparisons. For categorical variables, Pearson’s chi-square test was employed. A *P*-value for multiple testing was adjusted with the Benjamini–Hochberg false discovery rate (FDR) method, considering a significance level of *P* < 0.05.

#### Gut bacteriome analysis

Alpha diversity was assessed using observed features and Shannon’s index, with a focus on genera. Differences in alpha diversity between the study groups were analyzed using the Kruskal–Wallis test, and Benjamini–Hochberg method was used to account for multiple testing. Beta diversity was characterized using principal components analysis (PCA) with Bray–Curtis Dissimilarity at the genus level, and differences in beta diversity between the study groups were analyzed using Permutational Analysis of Variance (PERMANOVA) (vegan, v2.6-4) (Anderson, 2001). Differential abundance (DA) analysis was carried out using the ANOVA-Like Differential Expression tool for compositional data (ALDEx2, v1.30.0), which employs a compositionally consistent approach for DA testing using centered log-ratio transformations for data preprocessing (Fernandes *et al.*, 2014), and Analysis of Compositions of Microbiomes with Bias Correction (ANCOM-BC, v2.0.2), which explicitly tests hypotheses about differential absolute taxon abundance, corrects for sampling fraction bias, and considers data compositionality (Lin and Peddada, 2020). Benjamini–Hochberg method was used to account for the multiple testing. Figures were created with RStudio (version 2022.12.0.353 with R version 4.2.2, R Foundation for Statistical Computing, Vienna, Austria) using the ggplot2 tool (v.3.4.0) and GraphPad Prism (version 9.3.0, GraphPad Software, San Diego, CA, USA).

Machine learning (ML) algorithms facilitate the handling of large-scale microbiome compositional data and the identification of microbial features, which are relevant for classifying the trait of interest. Such approaches have successfully been used to build microbiome-based classification models for various complex diseases (Namkung, 2020; Liu *et al.*, 2022). The ML analyses were implemented using the scikit-learn package (Pedregosa *et al.*, 2011) and the q2-sample-classifier plugin. Given the unique advantages of handling various factors, such as the large number of microbial species, the abundance distribution, and the high-dimensional nature of the data itself, AdaBoost, Extra Trees, and RandomForest (RF) ensemble classifiers were utilized (Freund and Schapire, 1997; Breiman, 2001; Geurts *et al.*, 2006). The receiver operating characteristic (ROC) of the AUC was used to assess the performance of the models. Macro-averaging gives equal weight to each category, while micro-averaging gives equal weight to each sample during classification.

A partial correlation coefficient was calculated to examine the associations between the 10 most abundant taxa and common clinical characteristics related to MD and PCOS, while mitigating the potential influence of confounding factors, using Spearman’s correlation coefficient (ppcor, v.1.1) (Kim, 2015). The predominant taxa typically hold a more substantial presence within the gut microbial community, and examining these prevalent taxa can offer valuable insights into the comprehensive structure and composition of the microbiome.

## Results

### Clinical characteristics of the study subjects

The study population comprised 307 women who responded to both PCOS and MD criteria scales and provided a stool sample at 46 years. To confirm alterations in the gut bacteriome associated with MDs in our study cohort, as aligned with previous findings, we categorized the participants based on MDs: 264 women with no-MD and 43 with MD cases. The whole MD cases (i.e. control MD+PCOS MD) exhibited higher waist circumference (FDR = 0.02) and FABP2 levels (FDR = 0.02) compared to the whole no-MD cases (i.e. control no-MD+PCOS no-MD) (Table 1). We then compared the clinical characteristics of the participants based on both MDs and PCOS status, resulting in 84 PCOS no-MD, 18 PCOS MD, 180 control no-MD, and 25 control MD cases (Table 2). Notably, we observed higher levels of parameters related to glucose metabolisms, such as HOMA-IR (FDR = 0.03), fasting glucose (FDR = 0.01), and fasting insulin (FDR = 0.04) in the PCOS MD cases compared to the PCOS no-MD cases. There were no significant differences observed in the clinical characteristics among the control cases. Additionally, the prevalence of MDs was 17.65% in the PCOS group and 12.20% in the control group (FDR = 0.41) by Pearson’s chi-square test.

### Gut bacterial community profile and associations with MDs

**Figure 1.**
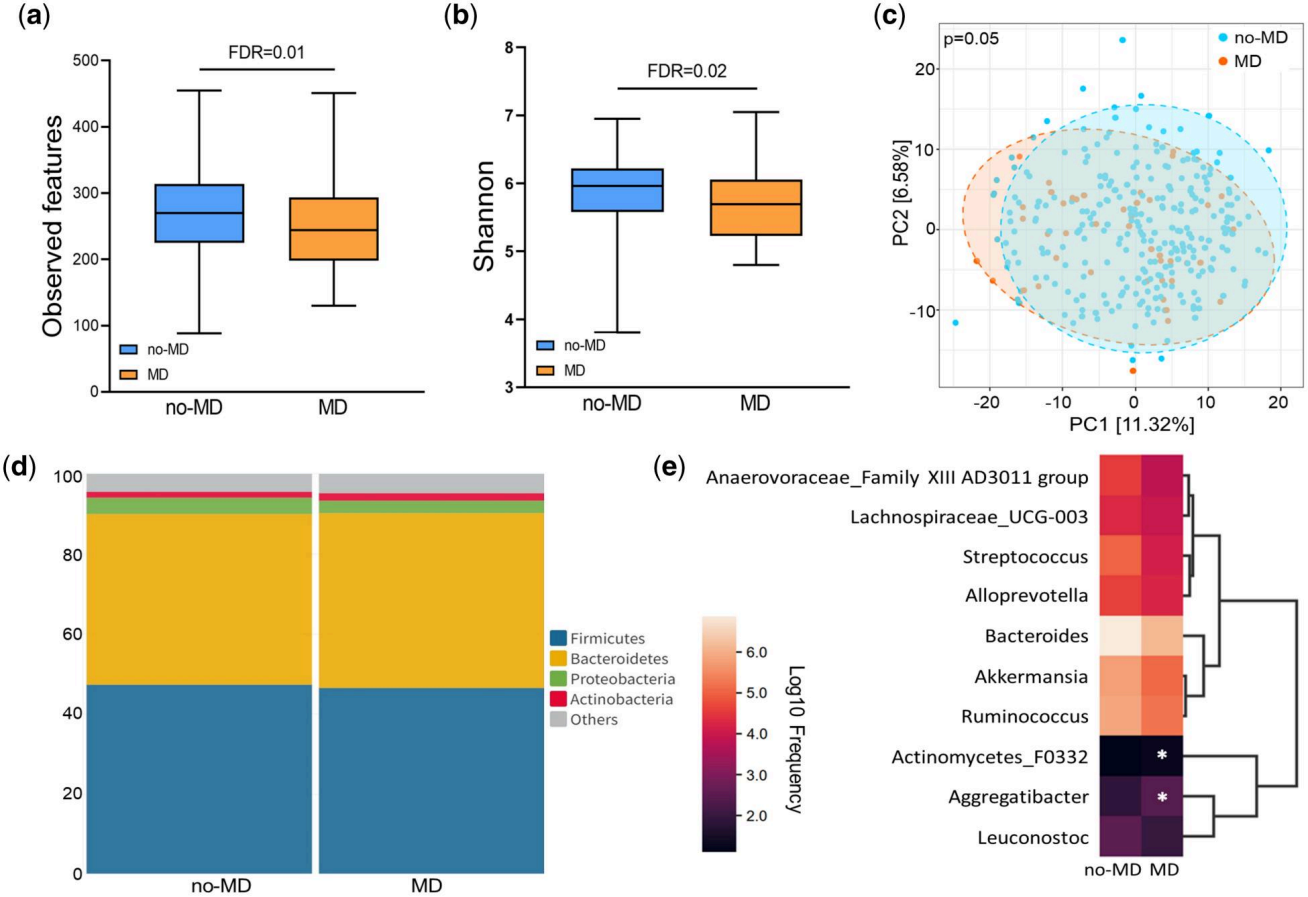
**Comparisons of the gut bacteriome in relation to MDs in the whole population.** The gut bacteriome profile of the subjects in the whole population, including both women with PCOS and control women, was analyzed. Alpha richness was analyzed using observed features and the Shannon index, and beta diversity was assessed using Bray–Curtis dissimilarity. In (**a**) observed features and (**b**) Shannon index. The box plots show the IQR, and the middle line represents the median values. Whiskers in the box plots denote minimum to maximum values. Blue represents no-MD and orange represents MD. A *P*-value was defined using the Kruskal–Wallis test adjusted with the Benjamini–Hochberg method. In the Bray–Curtis distances of (**c**) no-MD and MD cases, each dot represents a single individual, and the variation is shown by the percentages at the two axes. Blue represents no-MD and orange represents MD. A *P*-value was defined by PERMANOVA analysis. (**d**) The relative abundances of the four major phyla are represented as median values. The phyla with a relative abundance of <1% were grouped as others. (**e**) The bacterial features classifying the cases based on MDs were analyzed using the Extra-Trees model. The abundance of each feature (log10 frequency) is indicated by the color scale of the heatmap. A *P*-value was calculated by the Mann–Whitney *U*-test adjusted with the Benjamini–Hochberg method. FDR, false discovery rate; IQR, interquartile range; MD, mood disorder; PC, principal component; PERMANOVA, permutational analysis of variance.

We identified 111 genera across all the samples (n = 307), with *Bacteroides* being the dominant genus, followed by *Faecalibacterium* and *Alistipes in each population* (Supplementary Table S2). First, we investigated alterations in the gut bacteriome associated with MD status within the whole population. The whole MD cases exhibited a decreased alpha diversity (FDR_Observed features_=0.01, FDR_Shannon_=0.02) but no distinct differences in beta diversity compared to the whole no-MD cases, as determined by PERMANOVA (*r*^2^=0.01, *P* = 0.05) (Fig. 1a–c).

We then examined the bacterial composition in individuals with and without MDs considering both shared and distinct features. We explored shared features by calculating median values of the relative abundance of four main bacteria phyla and the top 10 most abundant genera in the study subjects. There were no significant differences in the median relative abundance between the no-MD and MD cases (Fig. 1d, Supplementary Table S3). Next, we assessed 126 taxa to identify distinctive features using ALDEx2 and ANCOM-BC analyses, but none of the taxa reached statistical significance after FDR adjustment (Supplementary Table S4). We then trained ML classifiers using the Extra-Trees model to distinguish cases based on MDs (AUC = 0.65) as both AdaBoost and RF models did not exceed an AUC of 0.6 (Supplementary Fig. S2). We identified 10 important features for the model, with statistically distinct relative frequencies in *Actinomycetes F0332* (FDR = 0.05) and *Aggregatibacter* (FDR = 0.04) between the no-MD and MD cases (Fig. 1e).

### The association of gut bacterial community with MDs in the PCOS group

**Figure 2.**
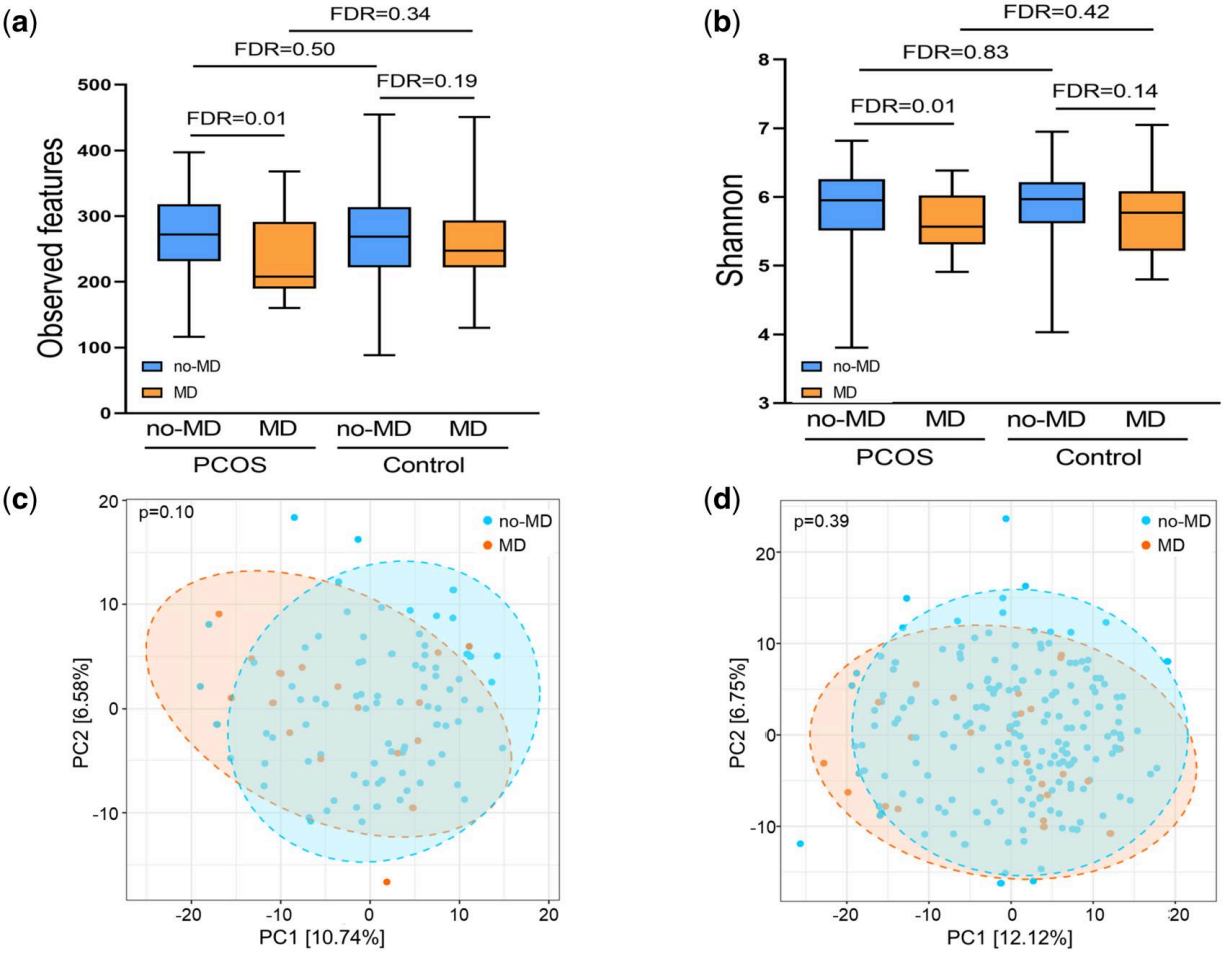
**Comparisons of the gut bacteriome in relation to MDs in the PCOS and control groups.** Alpha richness was analyzed using observed features and the Shannon index, and beta diversity was assessed using Bray–Curtis dissimilarity. In (**a**) observed features and (**b**) Shannon index, The box plots show the IQR, and the middle line represents the median values. Whiskers in the box plots denote minimum to maximum values. Blue represents no-MD and orange represents MD. A *P*-value was defined using the Kruskal–Wallis test adjusted with the Benjamini–Hochberg method. In the Bray–Curtis of (**c**) PCOS and (**d**) control groups, each dot represents a single individual, and the variation is shown by the percentages at the two axes. Blue represents no-MD and orange represents MD. A *P*-value was defined by PERMANOVA analysis. FDR, false discovery rate; IQR, interquartile range; MD, mood disorder; PC, principal component; PERMANOVA, permutational analysis of variance.

Next, we investigated the gut bacteriome alterations in relation to MDs in the presence of PCOS. Similar to the whole population, the PCOS MD cases showed lower alpha diversity than the PCOS no-MD cases (FDR_Observed features_=0.01, FDR_Shannon_=0.01) (Fig. 2a and b), and no difference in beta-diversity was observed (PERMANOVA; *r*^2^=0.01, *P* = 0.10) (Fig. 2c). Differences in diversity related to MDs could not be detected within the control group (FDR_Observed features_=0.19, FDR_Shannon_=0.14, as shown in Fig. 2a and b, PERMANOVA; *r*^2^=0.01, *P* = 0.39, as shown in Fig. 2d). Additionally, PCOS status did not influence community diversity when comparing cases with MDs (i.e. control MD vs PCOS MD), including both alpha diversity (FDR_Observed features_=0.34, FDR_Shannon_=0.42, as shown in Fig. 2a and b) and beta diversity (PERMANOVA; *r*^2^=0.02, *P* = 0.96, data not shown).

The most abundant phyla in the PCOS population are shown in Supplementary Fig. S3. In the comparisons of the median-based relative abundance of the 10 most abundant genera in the PCOS group, *Sutterella* showed a higher abundance in the PCOS MD cases compared to the PCOS no-MD cases (PCOS no-MD, median 0.00%; PCOS MD, median 0.14%, FDR < 0.001) (Table 3). In the DA analyses, while the control group showed no significant variation in relation to MDs (Supplementary Table S5), *Butyricicoccus* displayed a DA associated with MDs in the PCOS group, even after multiple testing corrections (ALDEx2, log-fold change (lfc)=−0.52, FDR = 0.06; ANCOM-BC, lfc=−0.90, FDR = 0.04) (Table 4). *Peptococcus* and *Agathobacter* showed DA between the control MD and PCOS MD cases in the ANCOM-BC analysis, but the significances were no longer present after multiple testing corrections (*Peptococcus*, FDR_ANCOM-BC_=0.99; *Agathobacter*, FDR_ANCOM-BC_=0.99).

**Table 3.** Relative abundance of the 10 most abundant genera in the PCOS group.

	PCOS		Control		FDR^a^		FDR^b^	
	no-MD (n = 84)	MD (n = 18)	no-MD (n = 180)	MD (n = 25)	PCOS	Control	no-MD	MD
*Bacteroides*	21.10 [14.62; 29.46]	27.06 [15.03; 36.73]	27.56 [16.19; 40.83]	24.65 [18.47; 37.92]	0.30	0.75	**0.00**	0.51
*Faecalibacterium*	11.35 [6.41; 18.61]	9.21 [5.09; 18.17]	13.33 [7.55; 19.42]	11.68 [8.17; 15.32]	0.55	0.24	0.18	0.56
*Alistipes*	8.25 [3.58; 13.45]	8.68 [4.13; 12.76]	10.62 [6.22; 16.59]	8.67 [4.60; 14.72]	0.82	0.35	**0.02**	0.77
*Parabacteroides*	1.30 [0.04; 3.76]	3.26 [0.41; 6.04]	1.07 [0.08; 4.49]	1.43 [0.30; 4.11]	0.22	0.85	0.60	0.44
*Ruminococcus*	1.59 [0.88; 4.05]	1.23 [0.59; 4.99]	1.67 [0.66; 3.43]	1.37 [0.54; 2.46]	0.72	0.31	0.46	0.51
*Subdoligranulum*	2.83 [1.10; 4.91]	1.55 [0.60; 4.56]	3.24 [1.37; 6.10]	2.47 [1.27; 3.80]	0.26	0.14	0.27	0.58
Oscillospiraceae_UCG_002	1.68 [0.40; 4.63]	1.78 [0.01; 2.98]	2.41 [0.60; 5.54]	1.65 [0.04; 7.39]	0.12	0.56	0.27	0.30
*Barnesiella*	1.57 [0.00; 4.62]	0.37 [0.00; 4.70]	0.57 [0.00; 3.01]	0.01 [0.00; 1.47]	0.99	0.12	0.09	0.14
*Sutterella*	0.00 [0.00; 0.00]	0.14 [0.13; 1.41]	0.00 [0.00; 0.45]	0.00 [0.00; 0.63]	**<0.001**	0.95	**0.00**	0.26
*Prevotella*	0.00 [0.00; 0.23]	0.00 [0.00; 0.01]	0.01 [0.00; 0.23]	0.00 [0.00; 3.60]	0.32	0.89	0.26	0.35

The 10 most abundant genera within the PCOS group are shown. The relative abundance of genera is presented as a median with an interquartile range [Q1; Q3]. *P*-value was determined by the Mann–Whitney *U*-test with the Benjamini–Hochberg FDR adjustment. FDR^a^ is the statistical analysis between no-MD and MD cases in each group (PCOS or control), and FDR^b^ is the statistical analysis between PCOS and control cases with the same mood disorder status. Bold values represent FDR < 0.05.

MD, mood disorder; FDR, false discovery rate.

**Table 4.** Differential abundant taxa between no-MD and MD cases in the PCOS group.

ALDEx2			ANCOM-BC		
Taxa	Effect size	FDR	Taxa	Effect size	FDR
*Butyricicoccus*	−0.52	0.06	*Butyricicoccus*	−0.90	**0.04**
Oscillospiraceae_uncultured	−0.55	0.09	Oscillospiraceae_uncultured	−0.72	0.47
*Holdemania*	−0.36	0.16	*Izemoplasmatales*	1.43	0.47
Ruminococcaceae_DTU089	−0.37	0.20	*Holdemania*	−1.23	0.47
*Oscillibacter*	−0.45	0.26	Ruminococcaceae_DTU089	−1.02	0.60
Ruminococcus_torques_group	−0.39	0.53	*Oscillibacter*	−0.52	0.60
*Bacteroides*	−0.37	0.54	Ruminococcus_torques_group	−0.54	0.60

The seven differentially abundant genera between no-MD and MD cases within the PCOS group are shown. A positive effect size value indicates a higher abundance of a taxon in the MD cases, while a negative value indicates a higher abundance in the no-MD cases. *P*-value was adjusted using the Benjamini–Hochberg method. Bold values represent FDR < 0.05.

MD, mood disorder; FDR, false discovery rate.

### Associations of the gut bacteriome in relation to common clinical features of PCOS and MDs

**Figure 3.**
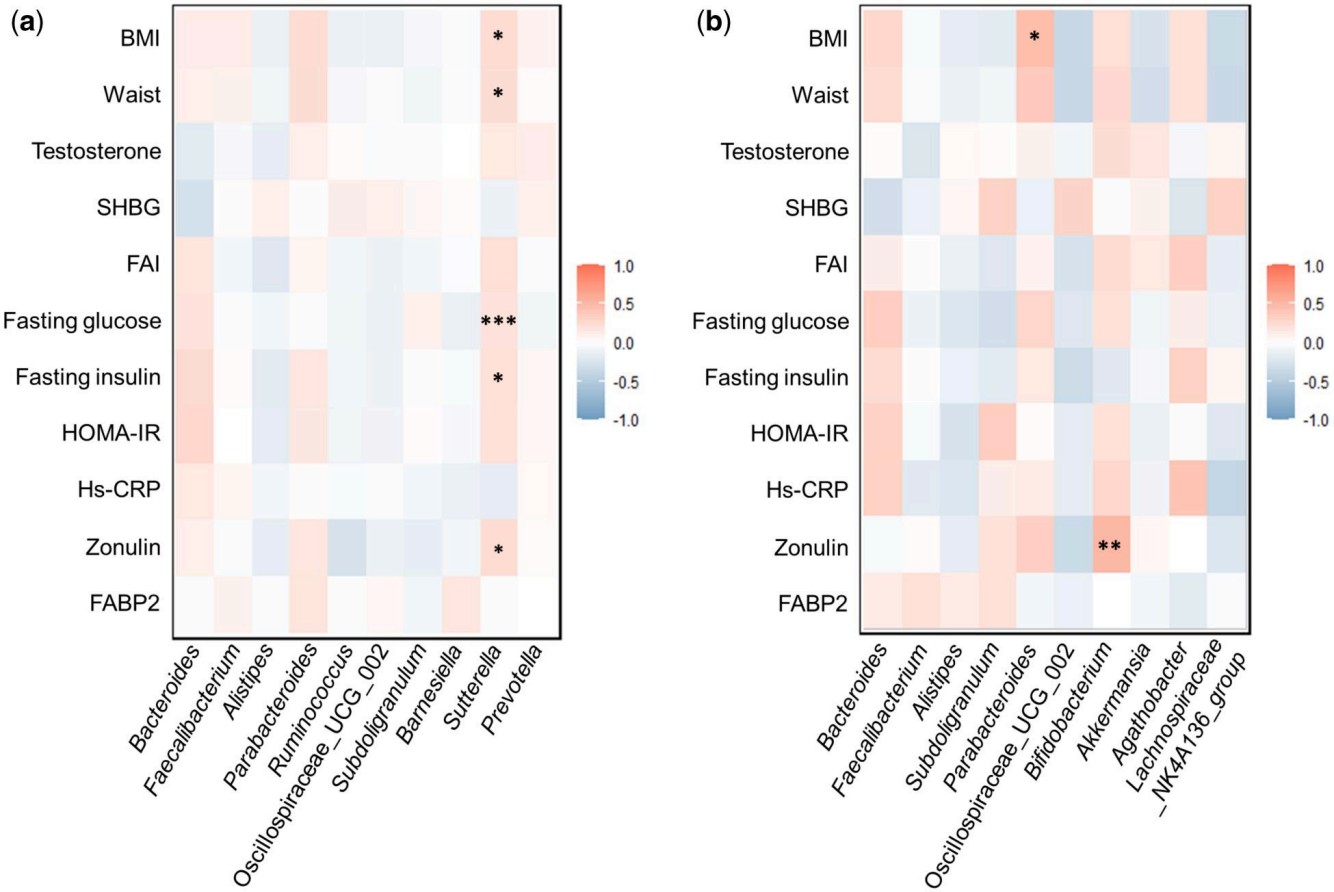
**The partial correlation of the top 10 most abundant genera with common clinical characteristics of PCOS and MDs.** Correlations between the 10 most abundant genera in (**a**) the PCOS group (i.e. PCOS no-MD+PCOS MD cases, n = 102), controlled by the presence of MDs, and (**b**) the MD group (i.e. control MD+PCOS MD, n = 43), controlled by the presence of PCOS. A negative correlation is indicated as blue and a positive correlation is indicated as red in the color key. A *P*-value was determined by the Kruskal–Wallis test adjusted with the Benjamini–Hochberg method. *FDR < 0.05, **FDR < 0.01, and ***FDR < 0.001. FDR, false discovery rate; MD, mood disorder; SHBG, sex hormone-binding globulin; FAI, free androgen index; HOMA-IR, homeostatic model assessment for insulin resistance; hs-CRP, high-sensitive C-reactive protein; FABP2, fatty acid-binding protein 2.

To explore potential connections between PCOS and MDs mediated by the gut bacteriome, we conducted partial correlation analyses between the most abundant genera and common clinical features of PCOS and MDs using two different study groups: (i) entire PCOS group (i.e. PCOS no-MD+PCOS MD) and (ii) entire MD group (i.e. control MD+PCOS MD) in order to mitigate their potential influence. In the PCOS group, *Sutterella* exhibited positive correlations with BMI (*r*^2^=0.31, FDR = 0.01), waist circumference (*r*^2^=0.29, FDR = 0.02), fasting glucose level (*r*^2^=0.46, FDR < 0.001), fasting insulin level (*r*^2^=0.24, FDR = 0.045), and zonulin (*r*^2^=0.25, FDR = 0.03) (Fig. 3a). Additionally, we observed positive correlations between *Parabacteroides* and BMI (*r*^2^=0.44, FDR = 0.02) and *Bifidobacterium* and zonulin level (*r*^2^=0.51, FDR = 0.01) in the MD group (Fig. 3b).

## Discussion

Previous studies have provided evidence that women diagnosed with PCOS are at higher risk for MDs compared to women without PCOS. However, currently, there is a lack of studies exploring the associations of the gut bacteriome with MDs in women with PCOS, even though the gut bacteriome can contribute to the underlying mechanisms of both disorders. Here we report, for the first time, the link between the gut bacteriome and MDs in PCOS.

Here, we observed decreased alpha diversity in the entire MD cases (i.e. control MD+PCOS MD) compared to the entire no-MD cases (i.e. control no-MD+PCOS no-MD), consistent with the previous findings (Simpson *et al.*, 2021), and identified *Actinomycetes F0332* and *Aggregatibacter* as features classifying subjects based on the presence or absence of MDs. Taking into account their associations with inflammation (Kamma *et al.*, 2004; Looh *et al.*, 2022; Miao *et al.*, 2022), in addition to our results of elevated FABP2 levels and a positive correlation between abundance of *Bifidobacterium* and zonulin across all of the MD cases, these findings are relevant given the literature on systemic inflammation resulting from gut barrier compromise and its role in MD development (Arneth, 2018; Peirce and Alviña, 2019; Simpson *et al.*, 2021).

Previous studies have reported (i) altered gut bacterial profiles and a decline in diversity among women with PCOS and their associations with clinical characteristics of PCOS (Lindheim *et al.*, 2017; Torres *et al.*, 2018; Jobira *et al.*, 2020; Lüll *et al.*, 2021), and (ii) compositional changes in the gut bacterial community and reduced diversity along with a decrease in short-chain fatty acids (SCFA) synthesis in MD patients (Huang *et al.*, 2018; Jiang *et al.*, 2018). Interestingly, here, we revealed a shift in gut bacterial diversity within the PCOS group in relation to MDs. These findings align with the general understanding that greater diversity corresponds to higher community stability and functionality, indicative of the beneficial ecological value of the gut bacterial system (Shade, 2017). Nevertheless, the precise causal relationship between PCOS and changes in the gut bacteriome remains unknown. It is also important to acknowledge that gut bacterial diversity associated with PCOS (Rizk and Thackray, 2021; Sola-Leyva *et al.*, 2023) and MDs (Järbrink-Sehgal and Andreasson, 2020) varies between studies. Indeed, we did not observe any differences in the gut bacteriome profiles between women with and without PCOS. This discrepancy could stem from variations in the criteria we utilize to select study subjects in the present study. Unlike previous studies, we consider criteria for both PCOS and MDs. Furthermore, similar gut bacteriome profiles between PCOS cases and controls could be explained by meticulously matching of age and BMI. Since these confounding factors can affect clinical features of PCOS (e.g. HA, IR) (Hsu, 2013; Moran *et al.*, 2015), MDs (Scott *et al.*, 2008; Faravelli *et al.*, 2013; Sharafi *et al.*, 2020; Gallagher *et al.*, 2023), and the microbiome (Maruvada *et al.*, 2017; Badal *et al.*, 2020; Walrath *et al.*, 2021; Sola-Leyva *et al.*, 2023), they should be carefully considered when comparing case and control groups. Additionally, it is likely that metrics, such as diversity indices, only offer a simplified view of the gut bacteriome and may not be sufficient to explain the complex crosstalk with the host (Simpson *et al.*, 2021).

The relative abundance of *Butyricicoccus* in the MD cases significantly differed from the no-MD cases within the PCOS group. *Butyricicoccus*, as a butyrate producer, can suppress inflammatory responses by regulating immune activation in the colon (Li *et al.*, 2018) and contribute to brain function by elevating the expression of brain-derived neurotrophic factor, known for its antidepressant-like effects and support of neuroplasticity (Zhou *et al.*, 2020b). Consistent with our findings, previous studies have reported a reduced abundance of *Butyricicoccus* in a PCOS-like rat model (Chang *et al.*, 2021), in GAD patients (Jiang *et al.*, 2018), and in women with depressive symptoms (Zhou *et al.*, 2020b; Takeda *et al.*, 2022).

These changes in the gut bacteriome may be linked to altered clinical features observed in the PCOS group. The PCOS MD cases showed altered glucose metabolism-related markers compared to the PCOS no-MD cases, thus supporting previous studies demonstrating associations between IR and MDs (Cooney *et al.*, 2017; Leonard and Wegener, 2020). Given that inflammation plays a role not only in insulin signaling (Caricilli and Saad, 2013; Leonard and Wegener, 2020) but also in the pathophysiology of PCOS (Shorakae *et al.*, 2018; Oróstica *et al.*, 2020) and MDs (Peirce and Alviña, 2019), it can be hypothesized that changes in the gut bacteriome and its metabolites in PCOS may trigger peripheral and local inflammation while simultaneously diminishing the synthesis of neurotransmitters like SCFAs, thereby contributing to neuroinflammation via vagal and spinal afferent neurons, ultimately impacting mood (Arneth, 2018; Simpson *et al.*, 2021).

Our findings demonstrated correlations between the relative abundance of *Sutterella* and PCOS-related clinical traits in the PCOS group. *Sutterella* adheres to host intestinal mucosal epithelium and appears to have pro-inflammatory functions (Hiippala *et al.*, 2016). Studies have shown associations between the relative abundance of *Sutterella* and (i) metabolic disorders (Lim *et al.*, 2017; Ferrocino *et al.*, 2018) and (ii) MDs (Williams *et al.*, 2012; Dong *et al.*, 2021) and a negative correlation has been reported between *Sutterella* and SCFAs, such as propionate and butyrate (Zhou *et al.*, 2023). Most importantly, women with PCOS and PCOS-like rodent models showed a higher abundance of *Sutterella* (Arroyo *et al.*, 2019; Zhou *et al.*, 2020a; Li *et al.*, 2022), and a reduction of SCFA-producing bacteria (Torres *et al.*, 2018; Zhang *et al.*, 2019; Chu *et al.*, 2020), which influence both inflammation (Peirce and Alviña, 2019; Silva *et al.*, 2020; He *et al.*, 2021) and the gut–brain axis (Lal *et al.*, 2001; Silva *et al.*, 2020). Negative associations between neurotransmitters (i.e. serotonin, ghrelin, and peptide YY) and serum T have also been detected (Liu *et al.*, 2017). However, there is conflicting evidence regarding *Sutterella* in relation to MDs (Dong *et al.*, 2021; Simpson *et al.*, 2021), as the virulence (Hiippala *et al.*, 2016) and function (Wang *et al.*, 2020; Zhou *et al.*, 2023) of *Sutterella* can vary among different species, limiting the interpretation of data obtained from animal studies.

The low ethnic and genetic variation of the study subjects and careful BMI-matching helped to minimize confounding factors. This study employed ML analysis as part of the methodologies, providing a data-driven approach to identify relevant patterns and relationships. Despite the strength of this study, discrepancies with previous research may arise from variations in study designs, including the sample size (Yurtdaş and Akdevelioğlu, 2020), sequencing methods (Lüll *et al.*, 2021), and criteria for defining MDs (Deeks *et al.*, 2011; Karjula *et al.*, 2017; Dokras *et al.*, 2018). For example, there are various psychiatric outcome measures, such as the Hospital Anxiety and Depression Scale, the State-Trait Anxiety Inventory, and the Center Hamilton depression rating scale, to assess MDs, apart from the criteria we used in this study (Dokras *et al.*, 2018). Thus, these discrepancies may lead to distinct categorizations of MDs, potentially impacting different landscapes of the gut bacteriome. Additionally, extended duration of sample storage after sample collection may influence sequencing outcomes, including changes in bacterial community diversity and composition (Shaw *et al.*, 2016; Ezzy *et al.*, 2019; Kim *et al.*, 2023), as well as bacterial functional stability (Kim *et al.*, 2023). While the integrity of DNA may degrade over time, even when stored at ultra-low temperatures like −80°C (Shaw *et al.*, 2016; Ezzy *et al.*, 2019), we mitigated the potential impact of prolonged sample storage through meticulous experimental protocols. For example, we collected samples within a short timeframe and stored them in the same freezer without any thawing (Cardona *et al.*, 2012; Gorzelak *et al.*, 2015). Furthermore, we employed advanced DNA extraction methodologies (Costea *et al.*, 2017), cutting-edge sequencing techniques (Caporaso *et al.*, 2012; Allali *et al.*, 2017), and enhanced error correction strategies using DADA2 (Callahan *et al.*, 2016). Focusing on a small segment of the 16S rRNA gene (V3–V4 region) instead of the entire genome or the full length of the 16S rRNA gene also helped mitigate potential DNA degradation, while still providing sufficient data for microbiome analysis (Cruz-Flores *et al.*, 2022). Even though the present study was the largest of its type to date, the number of women with PCOS stratified by the presence of MDs remains limited, thereby affecting the attainability of statistical power. Despite these limitations, our study was the first to elucidate alterations in the gut bacteriome in women with PCOS and MDs, spanning from the community level to the specific taxa level.

## Conclusion

Based on the study results, it appears that the women with co-occurring PCOS and MDs have a distinct gut bacteriome with reduced alpha diversity and a significantly lower abundance of *Butyricicoccus* compared to women with PCOS but without MDs. Notably, the relative abundance of *Sutterella* showed associations with common clinical features of both PCOS and MDs, particularly those related to obesity and gut barrier integrity. This study opens a new avenue for investigating the pathophysiology of MDs in the context of PCOS.

## Supplementary Material

deae073_Supplementary_Figure_S1

deae073_Supplementary_Figure_S2

deae073_Supplementary_Figure_S3

deae073_Supplementary_Table_S1

deae073_Supplementary_Table_S2

deae073_Supplementary_Table_S3

deae073_Supplementary_Table_S4

deae073_Supplementary_Table_S5

## Data Availability

The 16S RNA sequencing data were submitted in the Sequence Read Archive (SRA) (Reference No. PRJNA669650) (https://trace.ncbi.nlm.nih.gov/Traces/sra/?study=SRP287519  https://www.ncbi.nlm.nih.gov/ bioproject/669650). NFBC data are available from the University of Oulu, Infrastructure for Population Studies. Permission to use the data can be requested for research purposes via electronic material request portal. In the use of data, we follow the EU general data protection regulation (679/2016) and Finnish Data Protection Act. The use of personal data is based on cohort participant’s written informed consent at his/her latest follow-up study, which may cause limitations to its use. Please, contact NFBC project center (NFBCprojectcenter@oulu.fi) and visit the cohort website (www.oulu.fi/nfbc) for more information.

## References

[deae073-B1] Aboeldalyl S , James C, Seyam E, Ibrahim EM, Shawki HE-D, Amer S. The role of chronic inflammation in polycystic ovarian syndrome—a systematic review and meta-analysis. Int J Mol Sci 2021;22:2734. 33800490 10.3390/ijms22052734 PMC7962967

[deae073-B2] Allali I , Arnold JW, Roach J, Cadenas MB, Butz N, Hassan HM, Koci M, Ballou A, Mendoza M, Ali R et al A comparison of sequencing platforms and bioinformatics pipelines for compositional analysis of the gut microbiome. BMC Microbiol 2017;17:194. 28903732 10.1186/s12866-017-1101-8 PMC5598039

[deae073-B3] Anderson MJ. A new method for non‐parametric multivariate analysis of variance. Austral Ecol 2001;26:32–46.

[deae073-B4] Arneth BM. Gut–brain axis biochemical signalling from the gastrointestinal tract to the central nervous system: gut dysbiosis and altered brain function. Postgrad Med J 2018;94:446–452. 30026389 10.1136/postgradmedj-2017-135424

[deae073-B5] Arroyo P , Ho BS, Sau L, Kelley ST, Thackray VG. Letrozole treatment of pubertal female mice results in activational effects on reproduction, metabolism and the gut microbiome. PLoS One 2019;14:e0223274. 31568518 10.1371/journal.pone.0223274 PMC6768472

[deae073-B6] Azziz R , Carmina E, Dewailly D, Diamanti-Kandarakis E, Escobar-Morreale HF, Futterweit W, Janssen OE, Legro RS, Norman RJ, Taylor AE et al; Androgen Excess Society. Position statement: criteria for defining polycystic ovary syndrome: an Androgen Excess Society guideline. J Clin Endocrinol Metab 2006;91:4237–4245. 16940456 10.1210/jc.2006-0178

[deae073-B7] Badal VD , Vaccariello ED, Murray ER, Yu KE, Knight R, Jeste DV, Nguyen TT. The gut microbiome, aging, and longevity: a systematic review. Nutrients 2020;12:3759. 33297486 10.3390/nu12123759 PMC7762384

[deae073-B8] Batra M , Bhatnager R, Kumar A, Suneja P, Dang AS. Interplay between PCOS and microbiome: the road less travelled. Am J Rep Immunol 2022;88:e13580. 10.1111/aji.13580 35598286

[deae073-B9] Beck AT , Steer RA, Brown GK. Beck Depression Inventory (BDI-II). San Antonio, TX: Psychological Corp, 1996, 10.

[deae073-B10] Bolyen E , Rideout JR, Dillon MR, Bokulich NA, Abnet CC, Al-Ghalith GA, Alexander H, Alm EJ, Arumugam M, Asnicar F et al Reproducible, interactive, scalable and extensible microbiome data science using QIIME 2. Nat Biotechnol 2019;37:852–857. 31341288 10.1038/s41587-019-0209-9 PMC7015180

[deae073-B11] Breiman L. Random forests. Mach Learn 2001;45:5–32.

[deae073-B12] Callahan BJ , McMurdie PJ, Rosen MJ, Han AW, Johnson AJA, Holmes SP. DADA2: high-resolution sample inference from Illumina amplicon data. Nat Methods 2016;13:581–583. 27214047 10.1038/nmeth.3869 PMC4927377

[deae073-B13] Caporaso JG , Lauber CL, Walters WA, Berg-Lyons D, Huntley J, Fierer N, Owens SM, Betley J, Fraser L, Bauer M et al Ultra-high-throughput microbial community analysis on the Illumina HiSeq and MiSeq platforms. ISME J 2012;6:1621–1624. 22402401 10.1038/ismej.2012.8 PMC3400413

[deae073-B14] Cardona S , Eck A, Cassellas M, Gallart M, Alastrue C, Dore J, Azpiroz F, Roca J, Guarner F, Manichanh C. Storage conditions of intestinal microbiota matter in metagenomic analysis. BMC Microbiol 2012;12:158. 22846661 10.1186/1471-2180-12-158 PMC3489833

[deae073-B15] Caricilli A , Saad M. The role of gut microbiota on insulin resistance. Nutrients 2013;5:829–851. 23482058 10.3390/nu5030829 PMC3705322

[deae073-B16] Cerf-Bensussan N , Gaboriau-Routhiau V. The immune system and the gut microbiota: friends or foes? Nat Rev Immunol 2010;10:735–744. 20865020 10.1038/nri2850

[deae073-B17] Chang Z , Deng G, Shao Y, Xu D, Zhao Y, Sun Y, Zhang S, Hou R, Liu J. Shaoyao-Gancao decoction ameliorates the inflammation state in polycystic ovary syndrome rats via remodeling gut microbiota and suppressing the TLR4/NF-κB pathway. Front Pharmacol 2021;12:670054. 34054541 10.3389/fphar.2021.670054 PMC8155368

[deae073-B18] Chen F , Chen Z, Chen M, Chen G, Huang Q, Yang X, Yin H, Chen L, Zhang W, Lin H et al Reduced stress-associated FKBP5 DNA methylation together with gut microbiota dysbiosis is linked with the progression of obese PCOS patients. NPJ Biofilms Microbiomes 2021;7:60. 34267209 10.1038/s41522-021-00231-6 PMC8282850

[deae073-B19] Chu W , Han Q, Xu J, Wang J, Sun Y, Li W, Chen Z-J, Du Y. Metagenomic analysis identified microbiome alterations and pathological association between intestinal microbiota and polycystic ovary syndrome. Fertil Steril 2020;113:1286–1298.e4. 32482258 10.1016/j.fertnstert.2020.01.027

[deae073-B20] Cooney LG , Lee I, Sammel MD, Dokras A. High prevalence of moderate and severe depressive and anxiety symptoms in polycystic ovary syndrome: a systematic review and meta-analysis. Hum Reprod 2017;32:1075–1091. 28333286 10.1093/humrep/dex044

[deae073-B21] Costea PI , Zeller G, Sunagawa S, Pelletier E, Alberti A, Levenez F, Tramontano M, Driessen M, Hercog R, Jung F-E et al Towards standards for human fecal sample processing in metagenomic studies. Nat Biotechnol 2017;35:1069–1076. 28967887 10.1038/nbt.3960

[deae073-B22] Cruz-Flores R , López-Carvallo JA, Cáceres-Martínez J, Dhar AK. Microbiome analysis from formalin-fixed paraffin-embedded tissues: current challenges and future perspectives. J Microbiol Methods 2022;196:106476. 35490989 10.1016/j.mimet.2022.106476

[deae073-B23] Deeks AA , Gibson-Helm ME, Paul E, Teede HJ. Is having polycystic ovary syndrome a predictor of poor psychological function including anxiety and depression? Hum Reprod 2011;26:1399–1407. 21436137 10.1093/humrep/der071

[deae073-B24] Devaraj S , Hemarajata P, Versalovic J. The human gut microbiome and body metabolism: implications for obesity and diabetes. Clin Chem 2013;59:617–628. 23401286 10.1373/clinchem.2012.187617 PMC3974587

[deae073-B25] Dokras A , Stener-Victorin E, Yildiz BO, Li R, Ottey S, Shah D, Epperson N, Teede H. Androgen Excess- Polycystic Ovary Syndrome Society: position statement on depression, anxiety, quality of life, and eating disorders in polycystic ovary syndrome. Fertil Steril 2018;109:888–899. 29778388 10.1016/j.fertnstert.2018.01.038

[deae073-B26] Dong Z , Shen X, Hao Y, Li J, Li H, Xu H, Yin L, Kuang W. Gut microbiome: a potential indicator for differential diagnosis of major depressive disorder and general anxiety disorder. Front Psychiatry 2021;12:651536. 34589003 10.3389/fpsyt.2021.651536 PMC8473618

[deae073-B27] Escobar-Morreale HF. Polycystic ovary syndrome: definition, aetiology, diagnosis and treatment. Nat Rev Endocrinol 2018;14:270–284. 29569621 10.1038/nrendo.2018.24

[deae073-B28] Ezzy AC , Hagstrom AD, George C, Hamlin AS, Pereg L, Murphy AJ, Winter G. Storage and handling of human faecal samples affect the gut microbiome composition: a feasibility study. J Microbiol Methods 2019;164:105668. 31302202 10.1016/j.mimet.2019.105668

[deae073-B29] Faravelli C , Alessandra Scarpato M, Castellini G, Lo Sauro C. Gender differences in depression and anxiety: the role of age. Psychiatry Res 2013;210:1301–1303. 24135551 10.1016/j.psychres.2013.09.027

[deae073-B30] Fernandes AD , Reid JN, Macklaim JM, McMurrough TA, Edgell DR, Gloor GB. Unifying the analysis of high-throughput sequencing datasets: characterizing RNA-seq, 16S rRNA gene sequencing and selective growth experiments by compositional data analysis. Microbiome 2014;2:15. 24910773 10.1186/2049-2618-2-15 PMC4030730

[deae073-B31] Ferrocino I , Ponzo V, Gambino R, Zarovska A, Leone F, Monzeglio C, Goitre I, Rosato R, Romano A, Grassi G et al Changes in the gut microbiota composition during pregnancy in patients with gestational diabetes mellitus (GDM). Sci Rep 2018;8:12216. 30111822 10.1038/s41598-018-30735-9 PMC6093919

[deae073-B32] Freund Y , Schapire RE. A decision-theoretic generalization of on-line learning and an application to boosting. J Comput Syst Sci 1997;55:119–139.

[deae073-B33] Gallagher C , Waidyatillake N, Pirkis J, Lambert K, Cassim R, Dharmage S, Erbas B. The effects of weight change from childhood to adulthood on depression and anxiety risk in adulthood: a systematic review. Obes Rev 2023;24:e13566. 37062534 10.1111/obr.13566

[deae073-B34] García-Bernal D , García-Arranz M, Yáñez RM, Hervás-Salcedo R, Cortés A, Fernández-García M, Hernando-Rodríguez M, Quintana-Bustamante Ó, Bueren JA, García-Olmo D et al The current status of mesenchymal stromal cells: controversies, unresolved issues and some promising solutions to improve their therapeutic efficacy. Front Cell Dev Biol 2021;9:650664. 33796536 10.3389/fcell.2021.650664 PMC8007911

[deae073-B35] Geurts P , Ernst D, Wehenkel L. Extremely randomized trees. Mach Learn 2006;63:3–42.

[deae073-B36] Gorzelak MA , Gill SK, Tasnim N, Ahmadi-Vand Z, Jay M, Gibson DL. Methods for improving human gut microbiome data by reducing variability through sample processing and storage of stool. PLoS One 2015;10:e0134802. 26252519 10.1371/journal.pone.0134802 PMC4529225

[deae073-B37] Guze SB. Diagnostic and statistical manual of mental disorders, 4th ed. (DSM-IV). Am J Psychiatry 1995;152:1228.

[deae073-B38] Haudum C , Lindheim L, Ascani A, Trummer C, Horvath A, Münzker J, Obermayer-Pietsch B. Impact of short-term isoflavone intervention in polycystic ovary syndrome (PCOS) patients on microbiota composition and metagenomics. Nutrients 2020;12:1622. 32492805 10.3390/nu12061622 PMC7656308

[deae073-B39] He F , Li Y. Role of gut microbiota in the development of insulin resistance and the mechanism underlying polycystic ovary syndrome: a review. J Ovarian Res 2020;13:73. 32552864 10.1186/s13048-020-00670-3 PMC7301991

[deae073-B40] He S , Li H, Yu Z, Zhang F, Liang S, Liu H, Chen H, Lü M. The gut microbiome and sex hormone-related diseases. Front Microbiol 2021;12:711137. 34650525 10.3389/fmicb.2021.711137 PMC8506209

[deae073-B41] Hiippala K , Kainulainen V, Kalliomäki M, Arkkila P, Satokari R. Mucosal prevalence and interactions with the epithelium indicate commensalism of *Sutterella* spp. Front Microbiol 2016;7:1706. 27833600 10.3389/fmicb.2016.01706 PMC5080374

[deae073-B42] Hooper LV , Macpherson AJ. Immune adaptations that maintain homeostasis with the intestinal microbiota. Nat Rev Immunol 2010;10:159–169. 20182457 10.1038/nri2710

[deae073-B43] Hsu M-I. Changes in the PCOS phenotype with age. Steroids 2013;78:761–766. 23624031 10.1016/j.steroids.2013.04.005

[deae073-B44] Huang Y , Shi X, Li Z, Shen Y, Shi X, Wang L, Li G, Yuan Y, Wang J, Zhang Y et al Possible association of Firmicutes in the gut microbiota of patients with major depressive disorder. Neuropsychiatr Dis Treat 2018;14:3329–3337. 30584306 10.2147/NDT.S188340 PMC6284853

[deae073-B45] Järbrink-Sehgal E , Andreasson A. The gut microbiota and mental health in adults. Curr Opin Neurobiol 2020;62:102–114. 32163822 10.1016/j.conb.2020.01.016

[deae073-B46] Jiang H , Zhang X, Yu Z, Zhang Z, Deng M, Zhao J, Ruan B. Altered gut microbiota profile in patients with generalized anxiety disorder. J Psychiatr Res 2018;104:130–136. 30029052 10.1016/j.jpsychires.2018.07.007

[deae073-B47] Jobira B , Frank DN, Pyle L, Silveira LJ, Kelsey MM, Garcia-Reyes Y, Robertson CE, Ir D, Nadeau KJ, Cree-Green M. Obese adolescents with PCOS have altered biodiversity and relative abundance in gastrointestinal microbiota. J Clin Endocrinol Metab 2020;105:e2134–e2144. 31970418 10.1210/clinem/dgz263 PMC7147870

[deae073-B48] Kamma JJ , Giannopoulou C, Vasdekis VGS, Mombelli A. Cytokine profile in gingival crevicular fluid of aggressive periodontitis: influence of smoking and stress. J Clin Periodontol 2004;31:894–902. 15367195 10.1111/j.1600-051X.2004.00585.x

[deae073-B49] Karjula S , Arffman RK, Morin-Papunen L, Franks S, Järvelin M-R, Tapanainen JS, Miettunen J, Piltonen TT. A population-based follow-up study shows high psychosis risk in women with PCOS. Arch Womens Ment Health 2021;25:301–311. 34841466 10.1007/s00737-021-01195-4 PMC8921102

[deae073-B50] Karjula S , Morin-Papunen L, Auvinen J, Ruokonen A, Puukka K, Franks S, Järvelin M-R, Tapanainen JS, Jokelainen J, Miettunen J et al Psychological distress is more prevalent in fertile age and premenopausal women with PCOS symptoms: 15-year follow-up. J Clin Endocrinol Metab 2017;102:1861–1869. 28323926 10.1210/jc.2016-3863 PMC5470769

[deae073-B51] Kim JH , Jeon J-Y, Im Y-J, Ha N, Kim J-K, Moon SJ, Kim M-G. Long-term taxonomic and functional stability of the gut microbiome from human fecal samples. Sci Rep 2023;13:114. 36596832 10.1038/s41598-022-27033-w PMC9810722

[deae073-B52] Kim S. ppcor: an R package for a fast calculation to semi-partial correlation coefficients. Commun Stat Appl Methods 2015;22:665–674. 26688802 10.5351/CSAM.2015.22.6.665 PMC4681537

[deae073-B53] Kolhe JV , Chhipa AS, Butani S, Chavda V, Patel SS. PCOS and depression: common links and potential targets. Reprod Sci 2022;29:3106–3123. 34642910 10.1007/s43032-021-00765-2

[deae073-B54] Lal S , Kirkup AJ, Brunsden AM, Thompson DG, Grundy D. Vagal afferent responses to fatty acids of different chain length in the rat. Am J Physiol Gastrointest Liver Physiol 2001;281:G907–G915. 11557510 10.1152/ajpgi.2001.281.4.G907

[deae073-B55] Leonard BE , Wegener G. Inflammation, insulin resistance and neuroprogression in depression. Acta Neuropsychiatr 2020;32:1–9. 10.1017/neu.2019.17 31186075

[deae073-B56] Li G , Liu Z, Ren F, Shi H, Zhao Q, Song Y, Fan X, Ma X, Qin G. Alterations of gut microbiome and fecal fatty acids in patients with polycystic ovary syndrome in Central China. Front Microbiol 2022;13:911992. 35847083 10.3389/fmicb.2022.911992 PMC9283120

[deae073-B57] Li Z , Zhu H, Zhang L, Qin C. The intestinal microbiome and Alzheimer’s disease: a review. Animal Model Exp Med 2018;1:180–188. 30891563 10.1002/ame2.12033 PMC6388077

[deae073-B58] Liang Y , Ming Q, Liang J, Zhang Y, Zhang H, Shen T. Gut microbiota dysbiosis in polycystic ovary syndrome: association with obesity – a preliminary report. Can J Physiol Pharmacol 2020;98:803–809. 32150694 10.1139/cjpp-2019-0413

[deae073-B59] Lim MY , You HJ, Yoon HS, Kwon B, Lee JY, Lee S, Song Y-M, Lee K, Sung J, Ko G. The effect of heritability and host genetics on the gut microbiota and metabolic syndrome. Gut 2017;66:1031–1038. 27053630 10.1136/gutjnl-2015-311326

[deae073-B60] Lin H , Peddada SD. Analysis of compositions of microbiomes with bias correction. Nat Commun 2020;11:3514. 32665548 10.1038/s41467-020-17041-7 PMC7360769

[deae073-B61] Lindheim L , Bashir M, Münzker J, Trummer C, Zachhuber V, Leber B, Horvath A, Pieber TR, Gorkiewicz G, Stadlbauer V et al Alterations in gut microbiome composition and barrier function are associated with reproductive and metabolic defects in women with polycystic ovary syndrome (PCOS): a pilot study. PLoS One 2017;12:e0168390. 28045919 10.1371/journal.pone.0168390 PMC5207627

[deae073-B62] Liu R , Zhang C, Shi Y, Zhang F, Li L, Wang X, Ling Y, Fu H, Dong W, Shen J et al Dysbiosis of gut microbiota associated with clinical parameters in polycystic ovary syndrome. Front Microbiol 2017;8:324. 28293234 10.3389/fmicb.2017.00324 PMC5328957

[deae073-B63] Liu W , Fang X, Zhou Y, Dou L, Dou T. Machine learning-based investigation of the relationship between gut microbiome and obesity status. Microbes Infect 2022;24:104892. 34678464 10.1016/j.micinf.2021.104892

[deae073-B64] Looh SC , Soo ZMP, Wong JJ, Yam HC, Chow SK, Hwang JS. Aggregatibacter actinomycetemcomitans as the aetiological cause of rheumatoid arthritis: what are the unsolved puzzles? Toxins (Basel) 2022;14:50. 35051027 10.3390/toxins14010050 PMC8777676

[deae073-B65] Lüll K , Arffman RK, Sola-Leyva A, Molina NM, Aasmets O, Herzig K-H, Plaza-Díaz J, Franks S, Morin-Papunen L, Tapanainen JS et al The gut microbiome in polycystic ovary syndrome and its association with metabolic traits. J Clin Endocrinol Metab 2021;106:858–871. 33205157 10.1210/clinem/dgaa848

[deae073-B66] Maruvada P , Leone V, Kaplan LM, Chang EB. The human microbiome and obesity: moving beyond associations. Cell Host Microbe 2017;22:589–599. 29120742 10.1016/j.chom.2017.10.005

[deae073-B67] Matthews DR , Hosker JP, Rudenski AS, Naylor BA, Treacher DF, Turner RC. Homeostasis model assessment: insulin resistance and β-cell function from fasting plasma glucose and insulin concentrations in man. Diabetologia 1985;28:412–419. 3899825 10.1007/BF00280883

[deae073-B68] Miao Z , Du W, Xiao C, Su C, Gou W, Shen L, Zhang J, Fu Y, Jiang Z, Wang Z et al Gut microbiota signatures of long-term and short-term plant-based dietary pattern and cardiometabolic health: a prospective cohort study. BMC Med 2022;20:204. 35701845 10.1186/s12916-022-02402-4 PMC9199182

[deae073-B69] Moran LJ , Norman RJ, Teede HJ. Metabolic risk in PCOS: phenotype and adiposity impact. Trends Endocrinol Metab 2015;26:136–143. 25591984 10.1016/j.tem.2014.12.003

[deae073-B70] Namkung J. Machine learning methods for microbiome studies. J Microbiol 2020;58:206–216. 32108316 10.1007/s12275-020-0066-8

[deae073-B71] Oróstica L , Poblete C, Romero C, Vega M. Pro-inflammatory markers negatively regulate IRS1 in endometrial cells and endometrium from women with obesity and PCOS. Reprod Sci 2020;27:290–300. 32046436 10.1007/s43032-019-00026-3

[deae073-B72] Pedregosa F , Varoquaux G, Gramfort A, Michel V, Thirion B, Grisel O, Blondel M, Prettenhofer P, Weiss R, Dubourg V. Scikit-learn: machine learning in Python. J Mach Learn Res 2011;12:2825–2830.

[deae073-B73] Peirce JM , Alviña K. The role of inflammation and the gut microbiome in depression and anxiety. J Neurosci Res 2019;97:1223–1241. 31144383 10.1002/jnr.24476

[deae073-B74] Pellock SJ , Redinbo MR. Glucuronides in the gut: sugar-driven symbioses between microbe and host. J Biol Chem 2017;292:8569–8576. 28389557 10.1074/jbc.R116.767434 PMC5448086

[deae073-B75] Price MN , Dehal PS, Arkin AP. FastTree 2—approximately maximum-likelihood trees for large alignments. PLoS One 2010;5:e9490. 20224823 10.1371/journal.pone.0009490 PMC2835736

[deae073-B76] Quast C , Pruesse E, Yilmaz P, Gerken J, Schweer T, Yarza P, Peplies J, Glöckner FO. The SILVA ribosomal RNA gene database project: improved data processing and web-based tools. Nucleic Acids Res 2012;41:D590–D596. 23193283 10.1093/nar/gks1219 PMC3531112

[deae073-B77] Rantakallio P. The longitudinal study of the Northern Finland birth cohort of 1966. Paediatr Perinat Epidemiol 1988;2:59–88. 2976931 10.1111/j.1365-3016.1988.tb00180.x

[deae073-B78] Revised 2003 Consensus on Diagnostic Criteria and Long-Term Health Risks Related to Polycystic Ovary Syndrome (PCOS). Hum Reprod 2004;19:41–47. 14688154 10.1093/humrep/deh098

[deae073-B79] Rizk MG , Thackray VG. Intersection of polycystic ovary syndrome and the gut microbiome. J Endocr Soc 2021;5:bvaa177. 33381671 10.1210/jendso/bvaa177 PMC7757431

[deae073-B80] Scott KM , Korff M, Von Alonso J, Angermeyer M, Bromet EJ, Bruffaerts R, de Girolamo G, de Graaf R, Fernandez A, Gureje O et al Age patterns in the prevalence of DSM-IV depressive/anxiety disorders with and without physical co-morbidity. Psychol Med 2008;38:1659–1669. 18485262 10.1017/S0033291708003413 PMC2637812

[deae073-B81] Shade A. Diversity is the question, not the answer. ISME J 2017;11:1–6. 27636395 10.1038/ismej.2016.118 PMC5421358

[deae073-B82] Sharafi SE , Garmaroudi G, Ghafouri M, Bafghi SA, Ghafouri M, Tabesh MR, Alizadeh Z. Prevalence of anxiety and depression in patients with overweight and obesity. Obes Med 2020;17:100169.

[deae073-B83] Shaw AG , Sim K, Powell E, Cornwell E, Cramer T, McClure ZE, Li M-S, Kroll JS. Latitude in sample handling and storage for infant faecal microbiota studies: the elephant in the room? Microbiome 2016;4:40. 27473284 10.1186/s40168-016-0186-x PMC4967342

[deae073-B84] Shorakae S , Ranasinha S, Abell S, Lambert G, Lambert E, Courten B D, Teede H. Inter-related effects of insulin resistance, hyperandrogenism, sympathetic dysfunction and chronic inflammation in PCOS. Clin Endocrinol (Oxf) 2018;89:628–633. 29992612 10.1111/cen.13808

[deae073-B85] Silva YP , Bernardi A, Frozza RL. The role of short-chain fatty acids from gut microbiota in gut-brain communication. Front Endocrinol (Lausanne) 2020;11:25. 32082260 10.3389/fendo.2020.00025 PMC7005631

[deae073-B86] Simpson CA , Diaz-Arteche C, Eliby D, Schwartz OS, Simmons JG, Cowan CSM. The gut microbiota in anxiety and depression—a systematic review. Clin Psychol Rev 2021;83:101943. 33271426 10.1016/j.cpr.2020.101943

[deae073-B87] Sinikumpu S-P , Jokelainen J, Tasanen K, Timonen M, Huilaja L. Association between pruritus and psychosocial well-being: a population-based study among 6,809 subjects. Acta Derm Venereol 2023;103:adv00837. 36598159 10.2340/actadv.v103.2922 PMC9885286

[deae073-B88] Sola-Leyva A , Pérez-Prieto I, Molina NM, Vargas E, Ruiz-Durán S, Leonés-Baños I, Canha-Gouveia A, Altmäe S. Microbial composition across body sites in polycystic ovary syndrome: a systematic review and meta-analysis. Reprod Biomed Online 2023;47:129–150. 37208218 10.1016/j.rbmo.2023.03.016

[deae073-B89] Swidsinski A , Loening-Baucke V, Theissig F, Engelhardt H, Bengmark S, Koch S, Lochs H, Dorffel Y. Comparative study of the intestinal mucus barrier in normal and inflamed colon. Gut 2007;56:343–350. 16908512 10.1136/gut.2006.098160 PMC1856798

[deae073-B90] Takeda T , Yoshimi K, Kai S, Ozawa G, Yamada K, Hiramatsu K. Characteristics of the gut microbiota in women with premenstrual symptoms: a cross-sectional study. PLoS One 2022;17:e0268466. 35622782 10.1371/journal.pone.0268466 PMC9140228

[deae073-B91] Teede HJ , Tay CT, Laven JJE, Dokras A, Moran LJ, Piltonen TT, Costello MF, Boivin J, Redman LM, Boyle JA et al Recommendations from the 2023 international evidence-based guideline for the assessment and management of polycystic ovary syndrome. J Clin Endocrinol Metab 2023;108:2447–2469. 37580314 10.1210/clinem/dgad463 PMC10505534

[deae073-B92] Torres PJ , Siakowska M, Banaszewska B, Pawelczyk L, Duleba AJ, Kelley ST, Thackray VG. Gut microbial diversity in women with polycystic ovary syndrome correlates with hyperandrogenism. J Clin Endocrinol Metab 2018;103:1502–1511. 29370410 10.1210/jc.2017-02153 PMC6276580

[deae073-B93] Tremellen K , Pearce K. Dysbiosis of Gut Microbiota (DOGMA) – a novel theory for the development of polycystic ovarian syndrome. Med Hypotheses 2012;79:104–112. 22543078 10.1016/j.mehy.2012.04.016

[deae073-B94] Walrath T , Dyamenahalli KU, Hulsebus HJ, McCullough RL, Idrovo J-P, Boe DM, McMahan RH, Kovacs EJ. Age-related changes in intestinal immunity and the microbiome. J Leukoc Biol 2021;109:1045–1061. 33020981 10.1002/JLB.3RI0620-405RR PMC8139861

[deae073-B95] Wang C , Zhang H, Liu H, Zhang H, Bao Y, Di J, Hu C. The genus *Sutterella* is a potential contributor to glucose metabolism improvement after Roux-en-Y gastric bypass surgery in T2D. Diabetes Res Clin Pract 2020;162:108116. 32194221 10.1016/j.diabres.2020.108116

[deae073-B96] Williams BL , Hornig M, Parekh T, Lipkin WI. Application of novel PCR-based methods for detection, quantitation, and phylogenetic characterization of *Sutterella* species in intestinal biopsy samples from children with autism and gastrointestinal disturbances. mBio 2012;3:e00261–e00311. 22233678 10.1128/mBio.00261-11 PMC3252763

[deae073-B97] Ye D , Ma I, Ma TY. Molecular mechanism of tumor necrosis factor-α modulation of intestinal epithelial tight junction barrier. Am J Physiol Gastrointest Liver Physiol 2006;290:G496–G504. 16474009 10.1152/ajpgi.00318.2005

[deae073-B98] Yurtdaş G , Akdevelioğlu Y. A new approach to polycystic ovary syndrome: the gut microbiota. J Am Coll Nutr 2020;39:371–382. 31513473 10.1080/07315724.2019.1657515

[deae073-B99] Zhang J , Sun Z, Jiang S, Bai X, Ma C, Peng Q, Chen K, Chang H, Fang T, Zhang H. Probiotic *Bifidobacterium lactis* V9 regulates the secretion of sex hormones in polycystic ovary syndrome patients through the gut-brain axis. mSystems 2019;4:e00017–e00019. 31020040 10.1128/mSystems.00017-19 PMC6469956

[deae073-B100] Zhou L , Ni Z, Cheng W, Yu J, Sun S, Zhai D, Yu C, Cai Z. Characteristic gut microbiota and predicted metabolic functions in women with PCOS. Endocr Connect 2020a;9:63–73. 31972546 10.1530/EC-19-0522 PMC6993273

[deae073-B101] Zhou Y , Chen C, Yu H, Yang Z. Fecal microbiota changes in patients with postpartum depressive disorder. Front Cell Infect Microbiol 2020b;10:567268. 33134190 10.3389/fcimb.2020.567268 PMC7550660

[deae073-B102] Zhou Y , Zhang F, Mao L, Feng T, Wang K, Xu M, Lv B, Wang X. Bifico relieves irritable bowel syndrome by regulating gut microbiota dysbiosis and inflammatory cytokines. Eur J Nutr 2023;62:139–155. 35918555 10.1007/s00394-022-02958-0 PMC9899748

[deae073-B103] Zhu X , Li Y, Jiang Y, Zhang J, Duan R, Liu L, Liu C, Xu X, Yu L, Wang Q et al Prediction of gut microbial community structure and function in polycystic ovary syndrome with high low-density lipoprotein cholesterol. Front Cell Infect Microbiol 2021;11:665406. 34350129 10.3389/fcimb.2021.665406 PMC8326754

