# Supplementary material for: Gut bacteriome and mood disorders in women with PCOS
Source: Hum Reprod. 2024 Apr 13;39(6):1291–302. doi: 10.1093/humrep/deae073 (PMC11145006; doi:10.1093/humrep/deae073)
Supplement: deae073_Supplementary_Figure_S1 [file deae073_supplementary_figure_s1.pdf]

human reproduction

Human Reproduction, 2024, 1–1
https://doi.org/10.1093/humrep/deae073
Supplementary Data

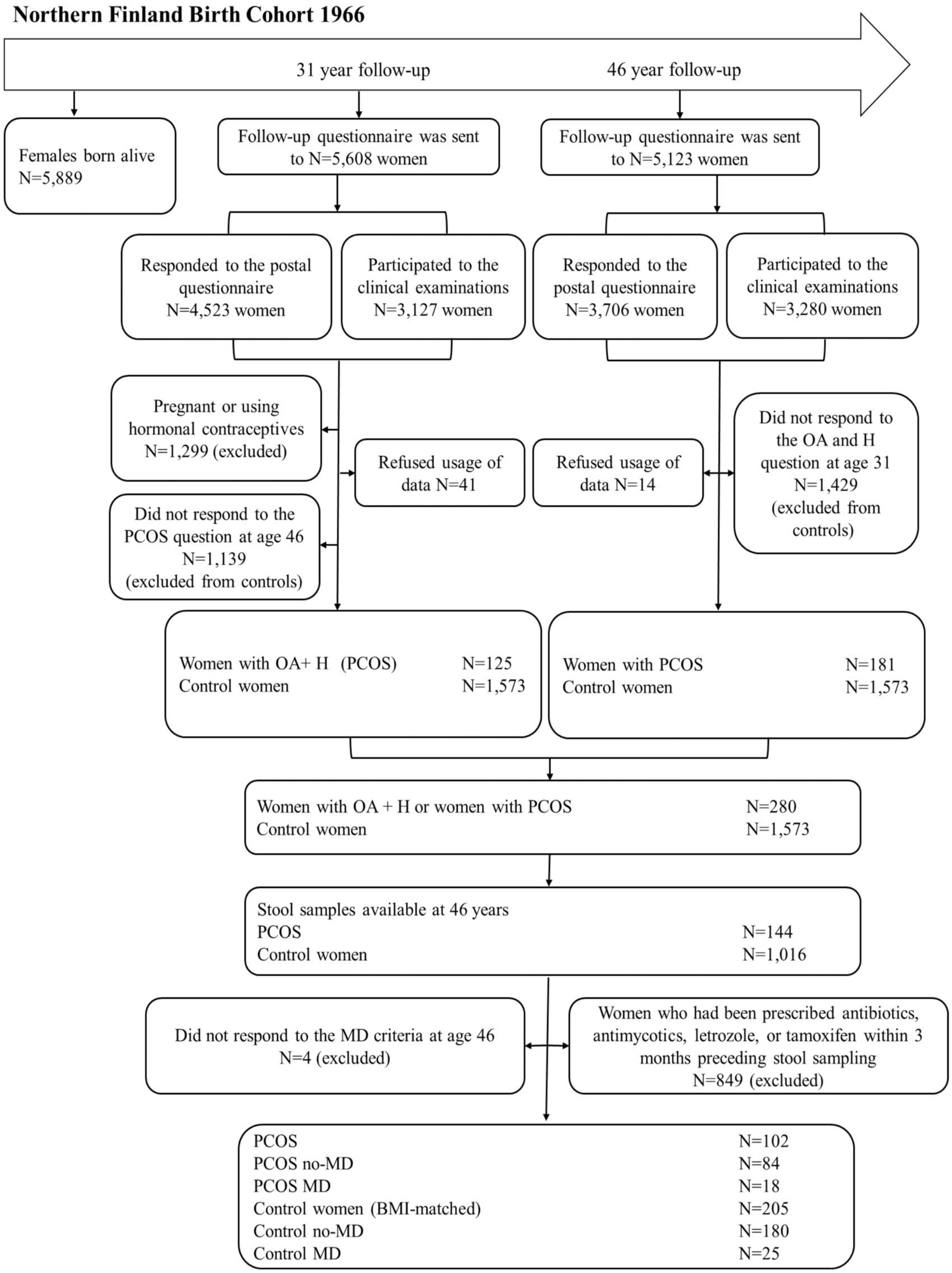

Supplementary Figure S1. Flowchart of the study population. MD, mood disorder; OA, oligo- or anovulation; H, hyperandrogenism.
