# Supplementary material for: Gut bacteriome and mood disorders in women with PCOS
Source: Hum Reprod. 2024 Apr 13;39(6):1291–302. doi: 10.1093/humrep/deae073 (PMC11145006; doi:10.1093/humrep/deae073)
Supplement: deae073_Supplementary_Figure_S2 [file deae073_supplementary_figure_s2.pdf]

OXFORD
human reproduction
Human Reproduction, 2024, 1–1
https://doi.org/10.1093/humrep/deae073
Supplementary Data

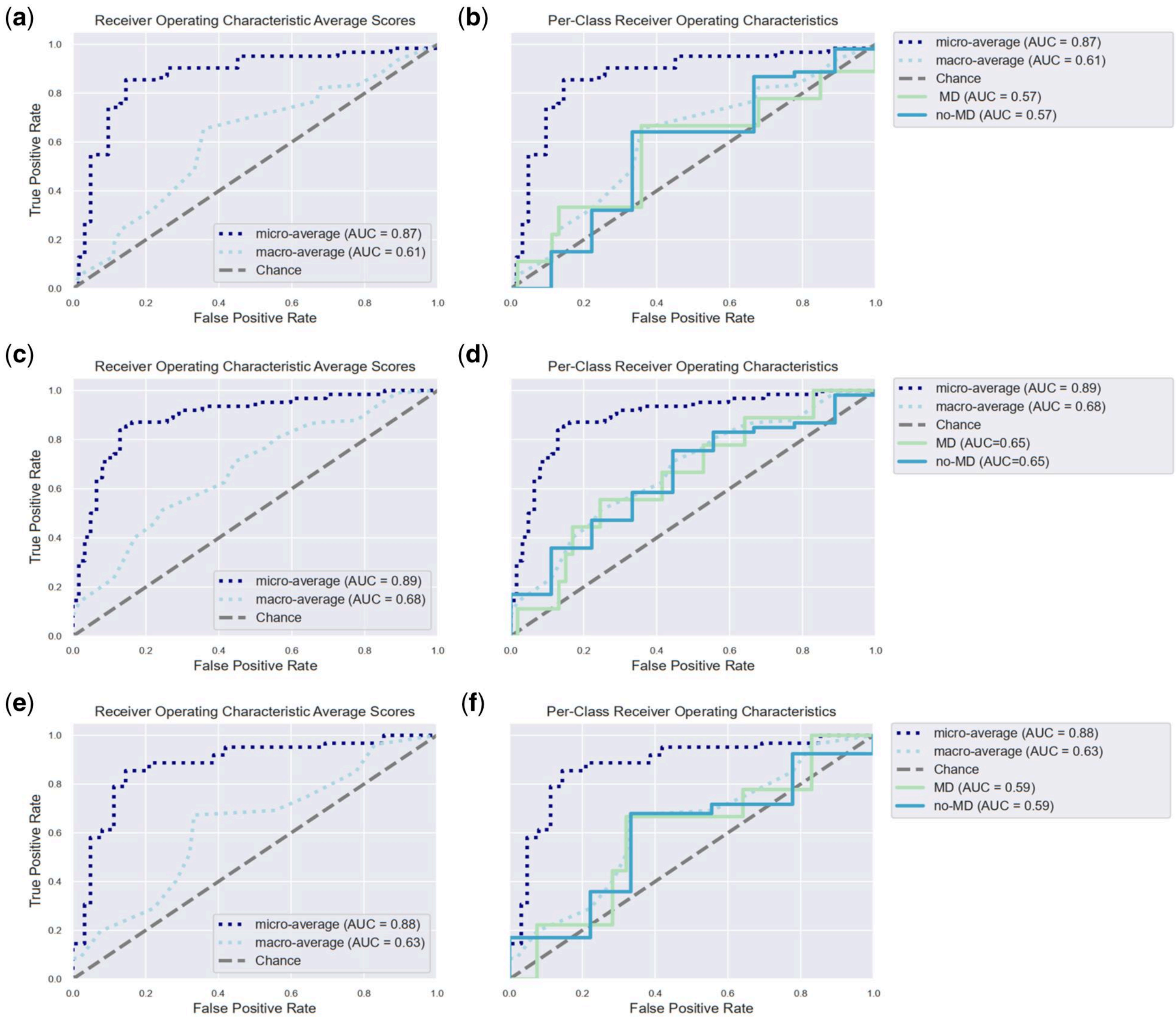

Supplementary Figure S2. Classifier performances predicting MDs using taxa abundances in the whole population. The receiver operating characteristics (ROC) curve of the real classifier is indicated by solid lines and random chance by dotted lines. The macro-averaging computes performance for each class and then finds the unweighted mean across all classes, while micro-averaging calculates the total counts of true positives, false positives, true negatives, and false negatives and then, using these counts, finds the average. no-MD samples are shown in blue lines and MD in green lines. (**a**, **b**) AdaBoost, (**c**, **d**) Extra-Trees, and (**e**, **f**) RandomForest. MD, mood disorder.
