# Supplementary material for: Gut bacteriome and mood disorders in women with PCOS
Source: Hum Reprod. 2024 Apr 13;39(6):1291–302. doi: 10.1093/humrep/deae073 (PMC11145006; doi:10.1093/humrep/deae073)
Supplement: deae073_Supplementary_Figure_S3 [file deae073_supplementary_figure_s3.pdf]

OXFORD
human reproduction
Human Reproduction, 2024, 1–1
https://doi.org/10.1093/humrep/deae073
Supplementary Data

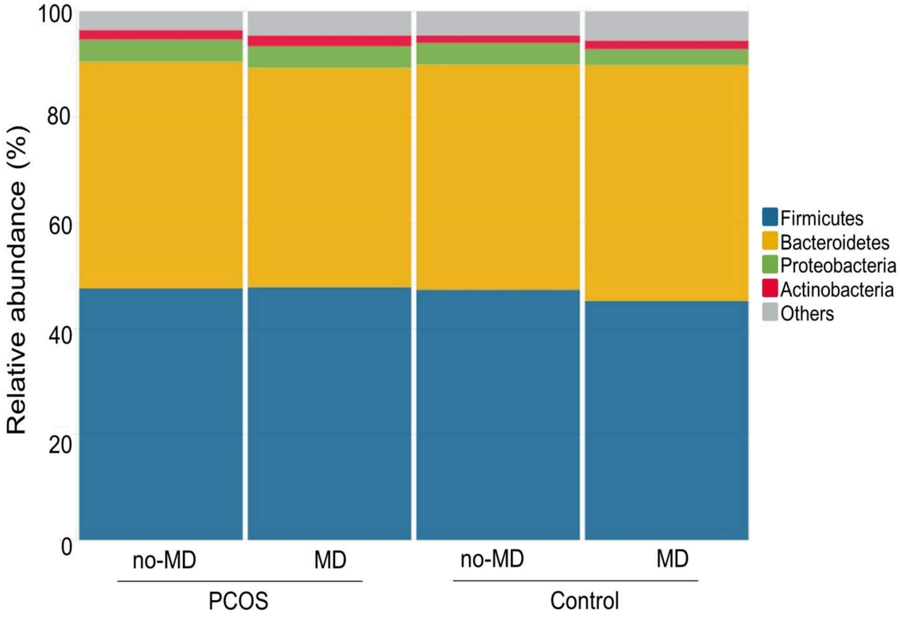

Supplementary Figure S3. Gut bacterial community at the phylum level in the PCOS and control group. The relative abundances of the major phyla are represented as median values. The phyla with a relative abundance of <1% were grouped as others. MD, mood disorder.
