# Supplementary material for: Gut bacteriome and mood disorders in women with PCOS
Source: Hum Reprod. 2024 Apr 13;39(6):1291–302. doi: 10.1093/humrep/deae073 (PMC11145006; doi:10.1093/humrep/deae073)
Supplement: deae073_Supplementary_Table_S1 [file deae073_supplementary_table_s1.pdf]

OXFORD
human reproduction

Human Reproduction, 2024, 1–1
https://doi.org/10.1093/humrep/deae073
Supplementary Data

Supplementary Table S1. Summary of no-MD and MD grouping criteria.

	BDI-II	GAD-7	HSCL-25		Self-reported diagnosis of depression
			Depression	Anxiety	
no-MD	≤14	≤5	≤1.55	≤1.55	No
MD	>14	>10	>1.75	>1.75	Yes

MD, mood disorder; BDI-II, Beck Depression Inventory Revision Version of the BDI; GAD-7, generalized anxiety disorder assessment-7; HSCL-25, Hopkins Symptom Checklist-25.
