# Supplementary material for: Gut bacteriome and mood disorders in women with PCOS
Source: Hum Reprod. 2024 Apr 13;39(6):1291–302. doi: 10.1093/humrep/deae073 (PMC11145006; doi:10.1093/humrep/deae073)
Supplement: deae073_Supplementary_Table_S2 [file deae073_supplementary_table_s2.pdf]

OXFORD
human reproduction

Human Reproduction, 2024, 1–3
https://doi.org/10.1093/humrep/deae073
Supplementary Data

Supplementary Table S2. Genera post-filtering with a prevalence exceeding 30%.

Phylum	Class	Order	Family	Genus	Prevalence	RA
Firmicutes	Bacilli	Erysipelotrichales	Erysipelotrichaceae	[Clostridium]_innocuum_group	100.0	24.6
Firmicutes	Clostridia	Oscillospirales	[Clostridium]_methylpentosum_group	[Clostridium]_methylpentosum_group	100.0	1.1
Firmicutes	Clostridia	Oscillospirales	[Eubacterium]_coprostanoligenes_group	[Eubacterium]_coprostanoligenes_group	100.0	0.4
Firmicutes	Clostridia	Lachnospirales	Lachnospiraceae	[Eubacterium]_eligens_group	100.0	0.9
Firmicutes	Clostridia	Lachnospirales	Lachnospiraceae	[Eubacterium]_hallii_group	99.7	9.5
Firmicutes	Clostridia	Oscillospirales	Ruminococcaceae	[Eubacterium]_siraeum_group	99.7	0.2
Firmicutes	Clostridia	Lachnospirales	Lachnospiraceae	[Eubacterium]_ventriosum_group	99.7	0.4
Firmicutes	Clostridia	Lachnospirales	Lachnospiraceae	[Eubacterium]_xylanophilum_group	99.3	7.4
Firmicutes	Clostridia	Lachnospirales	Lachnospiraceae	[Ruminococcus]_gauvreauii_group	99.3	0.3
Firmicutes	Clostridia	Lachnospirales	Lachnospiraceae	[Ruminococcus]_gnavus_group	99.3	0.2
Firmicutes	Clostridia	Lachnospirales	Lachnospiraceae	[Ruminococcus]_torques_group	99.3	0.3
Actinobacteriota	Coriobacteriia	Coriobacteriales	Eggerthellaceae	Adlercreutzia	99.3	0.2
Firmicutes	Clostridia	Lachnospirales	Lachnospiraceae	Agathobacter	99.0	0.9
Verrucomicrobiota	Verrucomicrobiae	Verrucomicrobiales	Akkermansiaceae	Akkermansia	98.7	0.3
Bacteroidota	Bacteroidia	Bacteroidales	Rikenellaceae	Alistipes	98.7	0.2
Firmicutes	Clostridia	Oscillospirales	Ruminococcaceae	Anaerofilum	98.7	1.1
Firmicutes	Clostridia	Lachnospirales	Lachnospiraceae	Anaerostipes	98.4	1.8
Firmicutes	Clostridia	Oscillospirales	Ruminococcaceae	Anaerotruncus	97.7	0.8
Bacteroidota	Bacteroidia	Bacteroidales	Bacteroidaceae	Bacteroides	97.7	0.1
Bacteroidota	Bacteroidia	Bacteroidales	Barnesiellaceae	Barnesiella	97.4	0.2
Actinobacteriota	Actinobacteria	Bifidobacteriales	Bifidobacteriaceae	Bifidobacterium	97.4	0.9
Desulfobacterota	Desulfovibrionia	Desulfovibrionales	Desulfovibrionaceae	Bilophila	97.4	0.1
Firmicutes	Clostridia	Lachnospirales	Lachnospiraceae	Blautia	97.1	0.1
Firmicutes	Clostridia	Oscillospirales	Butyricicoccaceae	Butyricicoccus	97.1	0.5
Bacteroidota	Bacteroidia	Bacteroidales	Marinifilaceae	Butyricimonas	96.7	0.3
Firmicutes	Clostridia	Lachnospirales	Lachnospiraceae	CAG-56	96.4	0.7
Firmicutes	Clostridia	Oscillospirales	Ruminococcaceae	Candidatus_Soleaferrea	96.4	1.1
Firmicutes	Clostridia	Christensenellales	Christensenellaceae	Christensenellaceae_R-7_group	96.1	0.1
Firmicutes	Clostridia	Clostridia_UCG-014	Clostridia_UCG-014	Clostridia_UCG-014	95.4	0.1
Firmicutes	Clostridia	Clostridia_vadinBB60_group	Clostridia_vadinBB60_group	Clostridia_vadinBB60_group	95.4	1.7
Firmicutes	Clostridia	Clostridiales	Clostridiaceae	Clostridium_sensu_stricto_1	94.8	0.5
Firmicutes	Clostridia	Oscillospirales	Oscillospiraceae	Colidextribacter	94.1	2.6
Actinobacteriota	Coriobacteriia	Coriobacteriales	Coriobacteriaceae	Collinsella	93.8	0.4
Bacteroidota	Bacteroidia	Bacteroidales	Barnesiellaceae	Coprobacter	93.8	0.3
Firmicutes	Clostridia	Lachnospirales	Lachnospiraceae	Coprococcus	93.8	2.1
Firmicutes	Clostridia	Lachnospirales	Defluviitaleaceae	Defluviitaleaceae_UCG-011	93.5	0.1
Firmicutes	Negativicutes	Veillonellales-Selenomonadales	Veillonellaceae	Dialister	91.8	0.4
Firmicutes	Bacilli	Erysipelotrichales	Erysipelotrichaceae	Dielma	91.2	0.0
Firmicutes	Clostridia	Lachnospirales	Lachnospiraceae	Dorea	90.5	0.3
Firmicutes	Incertae_Sedis	DTU014	DTU014	DTU014	89.9	0.3
Firmicutes	Clostridia	Oscillospirales	Ruminococcaceae	DTU089	89.2	0.1
Actinobacteriota	Coriobacteriia	Coriobacteriales	Eggerthellaceae	Eggerthella	88.9	0.0
Firmicutes	Clostridia	Lachnospirales	Lachnospiraceae	Eisenbergiella	87.6	0.0
Firmicutes	Bacilli	Erysipelotrichales	Erysipelatoclostridiaceae	Erysipelatoclostridium	87.3	0.6

(continued)

Supplementary Table S2. Continued

Phylum	Class	Order	Family	Genus	Prevalence	RA
Firmicutes	Bacilli	Erysipelotrichales	Erysipelatoclostridiaceae	Erysipelotrichaceae_UCG-003	86.9	0.5
Proteobacteria	Gammaproteobacteria	Enterobacterales	Enterobacteriaceae	Escherichia-Shigella	86.9	0.1
Firmicutes	Clostridia	Oscillospirales	Ruminococcaceae	Faecalibacterium	86.6	0.0
Firmicutes	Clostridia	Peptostreptococcales-Tissierellales	Anaerovoracaceae	Family_XIII_AD3011_group	85.6	0.0
Firmicutes	Clostridia	Peptostreptococcales-Tissierellales	Anaerovoracaceae	Family_XIII_UCG-001	85.3	0.4
Firmicutes	Clostridia	Oscillospirales	Oscillospiraceae	Flavonifractor	85.0	0.3
Firmicutes	Clostridia	Lachnospirales	Lachnospiraceae	Fusicatenibacter	85.0	0.0
Firmicutes	Clostridia	Lachnospirales	Lachnospiraceae	GCA-900066575	85.0	0.3
Firmicutes	Bacilli	Lactobacillales	Carnobacteriaceae	Granulicatella	84.6	0.0
Proteobacteria	Gammaproteobacteria	Pasteurellales	Pasteurellaceae	Haemophilus	83.7	0.0
Firmicutes	Bacilli	Erysipelotrichales	Erysipelotrichaceae	Holdemania	83.0	0.3
Firmicutes	Clostridia	Oscillospirales	Ruminococcaceae	Incertae_Sedis	82.7	0.0
Firmicutes	Clostridia	Peptostreptococcales-Tissierellales	Peptostreptococcaceae	Intestinibacter	82.7	0.1
Firmicutes	Clostridia	Oscillospirales	Oscillospiraceae	Intestinimonas	80.7	0.0
Firmicutes	Bacilli	Izemoplasmatales	Izemoplasmatales	Izemoplasmatales	80.4	0.1
Firmicutes	Clostridia	Lachnospirales	Lachnospiraceae	Lachnoclostridium	79.7	0.2
Firmicutes	Clostridia	Lachnospirales	Lachnospiraceae	Lachnospira	79.1	0.6
Firmicutes	Clostridia	Lachnospirales	Lachnospiraceae	Lachnospiraceae_FCS020_group	79.1	1.3
Firmicutes	Clostridia	Lachnospirales	Lachnospiraceae	Lachnospiraceae_ND3007_group	78.4	0.2
Firmicutes	Clostridia	Lachnospirales	Lachnospiraceae	Lachnospiraceae_NK4A136_group	78.1	0.0
Firmicutes	Clostridia	Lachnospirales	Lachnospiraceae	Lachnospiraceae_UCG-001	77.8	0.1
Firmicutes	Clostridia	Lachnospirales	Lachnospiraceae	Lachnospiraceae_UCG-004	77.8	0.0
Firmicutes	Clostridia	Lachnospirales	Lachnospiraceae	Lachnospiraceae_UCG-008	77.5	0.1
Firmicutes	Clostridia	Lachnospirales	Lachnospiraceae	Lachnospiraceae_UCG-010	77.1	0.0
Firmicutes	Bacilli	Lactobacillales	Lactobacillaceae	Lactobacillus	75.5	0.0
Firmicutes	Bacilli	Lactobacillales	Streptococcaceae	Lactococcus	73.5	0.0
Firmicutes	Clostridia	Lachnospirales	Lachnospiraceae	Marvinbryantia	73.2	0.0
Firmicutes	Bacilli	Erysipelotrichales	Erysipelotrichaceae	Merdibacter	71.9	0.4
Firmicutes	Clostridia	Monoglobales	Monoglobaceae	Monoglobus	71.2	0.0
Firmicutes	Clostridia	Lachnospirales	Lachnospiraceae	Moryella	70.3	0.0
Bacteroidota	Bacteroidia	Bacteroidales	Muribaculaceae	Muribaculaceae	69.9	0.7
Firmicutes	Clostridia	Oscillospirales	Ruminococcaceae	Negativibacillus	69.3	0.0
Firmicutes	Clostridia	Oscillospirales	Oscillospiraceae	NK4A214_group	69.0	0.0
Bacteroidota	Bacteroidia	Bacteroidales	Marinifilaceae	Odoribacter	67.6	0.0
Firmicutes	Clostridia	Oscillospirales	Oscillospiraceae	Oscillibacter	66.3	0.0
Firmicutes	Clostridia	Oscillospirales	Oscillospiraceae	Oscillospira	66.0	0.0
Firmicutes	Clostridia	Oscillospirales	Ruminococcaceae	Paludicola	64.7	0.0
Bacteroidota	Bacteroidia	Bacteroidales	Tannerellaceae	Parabacteroides	64.7	0.0
Bacteroidota	Bacteroidia	Bacteroidales	Prevotellaceae	Paraprevotella	64.1	0.0
Proteobacteria	Gammaproteobacteria	Burkholderiales	Sutterellaceae	Parasutterella	64.1	0.0
Firmicutes	Clostridia	Peptococcales	Peptococcaceae	Peptococcus	63.1	0.0
Firmicutes	Negativicutes	Acidaminococcales	Acidaminococcaceae	Phascolarctobacterium	62.4	0.0
Firmicutes	Clostridia	Oscillospirales	Ruminococcaceae	Phocea	61.4	0.0
Bacteroidota	Bacteroidia	Bacteroidales	Prevotellaceae	Prevotella	60.8	0.0
Firmicutes	Clostridia	Oscillospirales	Oscillospiraceae	Pseudoflavonifractor	60.1	0.0
Firmicutes	Bacilli	RF39	RF39	RF39	59.2	0.0
Firmicutes	Clostridia	Peptostreptococcales-Tissierellales	Peptostreptococcaceae	Romboutsia	58.2	0.0
Firmicutes	Clostridia	Lachnospirales	Lachnospiraceae	Roseburia	56.9	0.0
Firmicutes	Clostridia	Clostridia	Hungateiclostridiaceae	Ruminiclostridium	52.0	0.0
Firmicutes	Clostridia	Oscillospirales	Ruminococcaceae	Ruminococcus	51.6	0.0
Firmicutes	Clostridia	Lachnospirales	Lachnospiraceae	Sellimonas	51.0	0.0
Actinobacteriota	Coriobacteriia	Coriobacteriales	Eggerthellaceae	Senegalimassilia	50.3	0.0
Firmicutes	Clostridia	Lachnospirales	Lachnospiraceae	Shuttleworthia	48.4	0.0
Firmicutes	Bacilli	Lactobacillales	Streptococcaceae	Streptococcus	47.4	0.0
Firmicutes	Clostridia	Oscillospirales	Ruminococcaceae	Subdoligranulum	47.1	0.0

(continued)

Supplementary Table S2. Continued

Phylum	Class	Order	Family	Genus	Prevalence	RA
Proteobacteria	Gammaproteobacteria	Burkholderiales	Sutterellaceae	Sutterella	46.7	0.0
Patescibacteria	Saccharimonadia	Saccharimonadales	Saccharimonadaceae	TM7x	46.4	0.0
Firmicutes	Bacilli	Erysipelotrichales	Erysipelotrichaceae	Turicibacter	46.4	0.0
Firmicutes	Clostridia	Lachnospirales	Lachnospiraceae	Tyzzerella	45.8	0.0
Firmicutes	Clostridia	Oscillospirales	Ruminococcaceae	UBA1819	45.1	0.0
Firmicutes	Clostridia	Oscillospirales	Oscillospiraceae	UCG-002	44.8	0.0
Firmicutes	Clostridia	Oscillospirales	Oscillospiraceae	UCG-003	42.5	0.0
Firmicutes	Clostridia	Oscillospirales	Oscillospiraceae	UCG-005	42.2	0.0
Firmicutes	Clostridia	Oscillospirales	Butyricicoccaceae	UCG-009	42.2	0.0
Firmicutes	Clostridia	Oscillospirales	UCG-010	UCG-010	40.5	0.0
Firmicutes	Negativicutes	Veillonellales-Selenomonadales	Veillonellaceae	Veillonella	31.4	0.0
Verrucomicrobiota	Lentisphaeria	Victivallales	Victivallaceae	Victivallis	30.4	0.0

Prevalence and median-based relative abundance are presented as percentages (%).
RA, relative abundance.
