# Supplementary material for: Gut bacteriome and mood disorders in women with PCOS
Source: Hum Reprod. 2024 Apr 13;39(6):1291–302. doi: 10.1093/humrep/deae073 (PMC11145006; doi:10.1093/humrep/deae073)
Supplement: deae073_Supplementary_Table_S3 [file deae073_supplementary_table_s3.pdf]

OXFORD
human reproduction
Human Reproduction, 2024, 1–1
https://doi.org/10.1093/humrep/deae073
Supplementary Data

Supplementary Table S3. Relative abundance of the 10 most abundant genera in the whole population.

Taxa	no-MD	MD	FDR
Bacteroides	22.04 [13.11; 31.52]	25.04 [16.97; 36.64]	0.26
Faecalibacterium	10.84 [6.27; 15.92]	10.91 [6.60; 15.95]	0.93
Alistipes	8.18 [4.49; 13.04]	8.43 [4.39; 13.65]	0.76
Subdoligranulum	2.64 [1.10; 4.59]	2.10 [0.98; 3.99]	0.57
Oscillospiraceae_UCG-002	2.32 [0.52; 5.34]	1.65 [0.03; 4.12]	0.37
Lachnospiraceae_NK4A136_group	1.69 [0.75; 3.11]	1.09 [0.28; 2.64]	0.12
Blautia	1.63 [1.02; 2.63]	1.61 [1.04; 2.63]	0.76
Oscillibacter	1.43 [0.62; 2.34]	1.43 [0.74; 2.68]	0.76
Roseburia	1.18 [0.54; 2.25]	0.97 [0.39; 1.85]	0.57
Bifidobacterium	1.17 [0.39; 2.82]	0.77 [0.27; 3.61]	0.76

The 10 most abundant genera within the whole population are shown. The relative abundance of bacteria is presented as a median with an interquartile range [Q1; Q3]. *P*-value was determined by the Mann–Whitney *U*-test adjusted with the Benjamini–Hochberg method.
MD, mood disorder; FDR, false discovery rate.
