# Supplementary material for: Gut bacteriome and mood disorders in women with PCOS
Source: Hum Reprod. 2024 Apr 13;39(6):1291–302. doi: 10.1093/humrep/deae073 (PMC11145006; doi:10.1093/humrep/deae073)
Supplement: deae073_Supplementary_Table_S4 [file deae073_supplementary_table_s4.pdf]

Human Reproduction, 2024, 1–1
https://doi.org/10.1093/humrep/deae073
Supplementary Data

Supplementary Table S4. Differential abundance genera between no-MD and MD cases in the whole population.

ALDEx2			ANCOM-BC		
Taxa	Effect size	FDR	Taxa	Effect size	FDR
Ruminococcaceae_DTU089	−0.3	0.08	*Oscillibacter*	−0.52	0.25
Oscillospiraceae_uncultured	−0.34	0.09	Ruminococcaceae_DTU089	−0.8	0.25
Holdemania	−0.25	0.12	Lachnospiraceae_UCG-008	0.65	0.25
Lachnospiraceae_uncultured	−0.28	0.13	*Holdemania*	−0.78	0.25
Bacteroidaceae	−0.3	0.13	Ruminococcus_torques_group	−0.52	0.25
Oscillospiraceae_uncultured	−0.26	0.14	*Anaerotruncus*	−0.61	0.4
Anaerotruncus	−0.22	0.16	*Butyricicoccus*	−0.45	0.4
Butyricicoccus	−0.23	0.17	*Erysipelatoclostridium*	−0.64	0.4
Haemophilus	−0.26	0.19	*Bacteroides*	−0.31	0.4
Sellimonas	−0.21	0.21	Candidatus_Soleaferrea	−0.54	0.4
Candidatus_Soleaferrea	−0.2	0.23	Oscillospiraceae_uncultured	−0.39	0.4
Erysipelatoclostridium	−0.2	0.25	Eubacterium_coprostanoligenes_group	0.69	0.4
Colidextribacter	−0.21	0.27	*Streptococcus*	0.57	0.4
Lachnospiraceae_UCG-008	−0.25	0.24	*Romboutsia*	0.61	0.4

The 14 differentially abundant genera between no-MD and MD cases in the whole population are shown. A positive effect size value indicates a higher abundance of a taxon in the MD cases, while a negative value indicates a higher abundance in the no-MD cases. P-value was adjusted using the Benjamini–Hochberg method. MD, mood disorder; FDR, false discovery rate.
