# Supplementary material for: Gut bacteriome and mood disorders in women with PCOS
Source: Hum Reprod. 2024 Apr 13;39(6):1291–302. doi: 10.1093/humrep/deae073 (PMC11145006; doi:10.1093/humrep/deae073)
Supplement: deae073_Supplementary_Table_S5 [file deae073_supplementary_table_s5.pdf]

OXFORD
human reproduction

Human Reproduction, 2024, 1–1
https://doi.org/10.1093/humrep/deae073
Supplementary Data

Supplementary Table S5. Differential abundance genera between no-MD and MD cases in the control group.

ALDEx2			ANCOM-BC		
**Taxa**	**Effect size**	**FDR**	**Taxa**	**Effect size**	**FDR**
Anaerotruncus	−0.28	0.6	*Lachnospiraceae_UCG-008*	0.91	0.48
Lachnospiraceae_UCG-008	0.33	0.57	*Streptococcus*	0.88	0.95
Ruminococcaceae_DTU089	−0.24	0.62	*Eubacterium_coprostanoligenes_group*	1.01	0.95
Oscillospiraceae_uncultured	−0.28	0.62	*Romboutsia*	0.85	0.95
[Clostridium]_methylpentosum_group	−0.21	0.62	*Anaerotruncus*	−0.64	0.95

The five differentially abundant genera between no-MD and MD cases in the control group are shown. A positive effect size value indicates a higher abundance of a taxon in the MD cases, while a negative value indicates a higher abundance in the no-MD cases. *P*-value was adjusted using the Benjamini–Hochberg method. MD, mood disorder; FDR, false discovery rate.
